# Ethylene signals through an ethylene receptor to modulate biofilm formation and root colonization in a beneficial plant-associated bacterium

**DOI:** 10.1371/journal.pgen.1011587

**Published:** 2025-02-07

**Authors:** T. Scott Carlew, Eric Brenya, Mahbuba Ferdous, Ishita Banerjee, Lauren Donnelly, Eric Heinze, Josie King, Briana Sexton, Randy F. Lacey, Arkadipta Bakshi, Gladys Alexandre, Brad M. Binder

**Affiliations:** 1 Department of Biochemistry & Cellular and Molecular Biology, University of Tennessee Knoxville, Knoxville, Tennessee, United States of America; 2 Genome Science and Technology Program, University of Tennessee, Knoxville, Tennessee, United States of America; Michigan State University, UNITED STATES OF AMERICA

## Abstract

Ethylene is a plant hormone involved in many aspects of plant growth and development as well as responses to stress. The role of ethylene in plant-microbe interactions has been explored from the perspective of plants. However, only a small number of studies have examined the role of ethylene in microbes. We demonstrated that *Azospirillum brasilense* contains a functional ethylene receptor that we call Azospirillum Ethylene Response1 (AzoEtr1) after the nomenclature used in plants. AzoEtr1 directly binds ethylene with high affinity. Treating cells with ethylene or disrupting the receptor reduces biofilm formation and colonization of plant root surfaces. Additionally, RNA sequencing and untargeted metabolomics showed that ethylene causes wide-spread metabolic changes that affect carbon and nitrogen metabolism. One result is the accumulation of poly-hydroxybutyrate. Our data suggests a model in which ethylene from host plants alters the density of colonization by *A. brasilense* and re-wires its metabolism, suggesting that the bacterium implements an adaptation program upon sensing ethylene. These data provide potential new targets to regulate beneficial plant-microbe interactions.

## Author summary

Food production is becoming a major concern because of reduced arable land from an increased human population and climate change. Application of beneficial microbes to plants is being explored to increase crop productivity. However, this approach often underperforms in field conditions. To better sustain beneficial plant-microbe associations, we must understand what controls the formation and maintenance of these associations. *Azospirillum brasilense* is a soil bacterium that associates with plant roots and is used as a bioinoculant to boost plant growth and stress tolerance. We provide evidence that *A. brasilense* contains a functional receptor for the gaseous plant hormone ethylene. Application of ethylene regulates biofilm formation and root colonization of several plant hosts by *A. brasilense*. Additionally, ethylene causes wide-spread metabolic changes in *A. brasilense*. This is the first study to demonstrate that a beneficial soil bacterium contains a functional ethylene receptor. These findings suggest that ethylene may act as a cross-kingdom signaling molecule from plants to bacteria that contain these ethylene receptors thereby identifying a potential target to potentially improve beneficial plant-microbe interactions.

## Introduction

Food security is an increasing concern due to climate change and an increasing human population. Future food security will require sustainable agricultural practices that ensure elevated crop productivity while reducing inputs from chemical fertilizers, particularly, nitrogen, which can have adverse environmental effects. One of several strategies to decrease the use of chemical fertilizers is to inoculate crops with beneficial microorganisms. The establishment of plant-microbe associations in the rhizosphere is receiving increasing attention because of its profound effect on plant growth and vigor. The so-called “rhizosphere effect”, which describes the enhanced activity and density of microbes around the roots compared to the surrounding soil, plays a critical role in shaping the microbial rhizosphere communities which directly impact plant health and stress tolerance [1–4]. Root exudates not only support the growth and activity of rhizosphere microbial communities by providing nutrients, they also select for the microbiome and mediate microbe-microbe and plant-microbe interactions [1,4–6]. The relationship between the microbiome and root exudates is bidirectional, as plant immune responses and phytohormone signaling can alter root exudate profiles [7–11].

Manipulating ethylene signaling or synthesis in plants or the application of ethylene can affect plant health as well as the population of microbes associated with the plant [12–18]. Ethylene was first discovered to be a phytohormone over 100 years ago and was the first identified gaseous, biological signaling molecule [19]. In plants, it controls and modulates many developmental and physiological processes and responses to various environmental cues and stressors [20]. It is naturally found in soil and depending on conditions, such as compaction and water saturation, can reach concentrations above 10 ppm, which is well above the levels needed to elicit responses in plants [20–23]. There are multiple sources of ethylene in the soil, including biotic biosynthesis by plants, bacteria, and fungi, as well as abiotic sources such as photochemical production triggered when sunlight is absorbed by dissolved organics [20,21]. Almost all research on ethylene as a signaling molecule has focused on its role as a plant hormone and the signal transduction pathway by which it is perceived by plants [24–26]. However, responses to ethylene are not limited to plants, and have been reported in bacteria, fungi, slime molds, marine sponges, and even human cell lines [27].

Plants are believed to have gained ethylene receptors and other two-component like receptors from the cyanobacterium that is the ancestor of the chloroplast [28–30]. In support of this, many cyanobacteria contain genes that encode proteins predicted to have an ethylene-binding domain and show saturable ethylene binding [29,31,32]. Additionally, phylogenetic analysis has shown that plant ethylene receptors cluster with cyanobacterial proteins predicted to have ethylene-binding domains [33]. One such cyanobacterium, *Synechocystis* sp. PCC 6803 (hereafter referred to as *Synechocystis*), contains a bifunctional receptor called Synechocystis ethylene response1 (SynEtr1) for both ethylene and light with a signaling pathway that includes a down-stream response regulator protein and a small non-coding RNA [32,34,35]. Unlike in plants, signaling from SynEtr1 relies on histidine kinase activity and phosphorelay to a response regulator protein [32,36]. In this species, ethylene causes various molecular and physiological changes including changes in the cell surface leading to increased biofilm formation and phototaxis [32,37–40].

Phylogenetic analyses indicate that putative ethylene receptors are found in other bacterial phyla, including many non-pathogenic proteobacteria that associate with plants [27,32,41]. This suggests that ethylene may be perceived by these bacteria to mediate their interactions with plant hosts. Most published studies examining the role of ethylene in plant-microbe associations have focused on microbe-induced changes in ethylene levels or signaling in plants [16,42–44]. Direct evidence for saturable ethylene binding to a specific receptor in microbes affecting physiology has only been obtained in *Synechocystis* and the arbuscular mycorrhizal fungus *Rhizophagus irregularis* [32,45].

A plant-associated bacterium, *Azospirillum brasilense*, contains a gene encoding a putative ethylene receptor [32]. We call this gene *Azospirillum ethylene response1* (*Azoetr1*) following the nomenclature used in plants. Bacteria of the genus *Azospirillum* are ubiquitous motile soil diazotrophic bacteria capable of colonizing the roots of a wide range of plants where they live as commensals [46]. *A. brasilense* Sp7 colonizes root surfaces and is used agriculturally to boost crop production [46]. While the plant growth-promoting effects of *A. brasilense* have been described in most detail in cereals, the beneficial association of *A. brasilense* with plants is not restricted to cereals since they have been reported in many plant species across botanical families [46] including *Solanum lycopersicum* (tomato) and *Arabidopsis thaliana* [47,48].

Inoculation of plants with *A. brasilense* alters the expression of genes in the plant related to ethylene biosynthesis and signaling [47,49–51]. However, nothing is known about the responses of *A. brasilense* to ethylene and the role of the putative ethylene receptor protein. This prompted us to examine the biological role of ethylene as a signal in this species and study how ethylene perception by the bacterium affects various traits including root colonization. Our research demonstrated that AzoEtr1 is a functional ethylene receptor that affects carbon and nitrogen metabolism. Our data suggest a model in which ethylene from host plants affects the pattern and density of colonization by *A. brasilense*. Because other plant-associated bacteria are predicted to contain ethylene receptors [27,32,41], this could represent a mechanism applicable to other bacteria.

## Results

### *A. brasilense* contains a functional ethylene-binding protein, AzoEtr1

**Fig 1:**
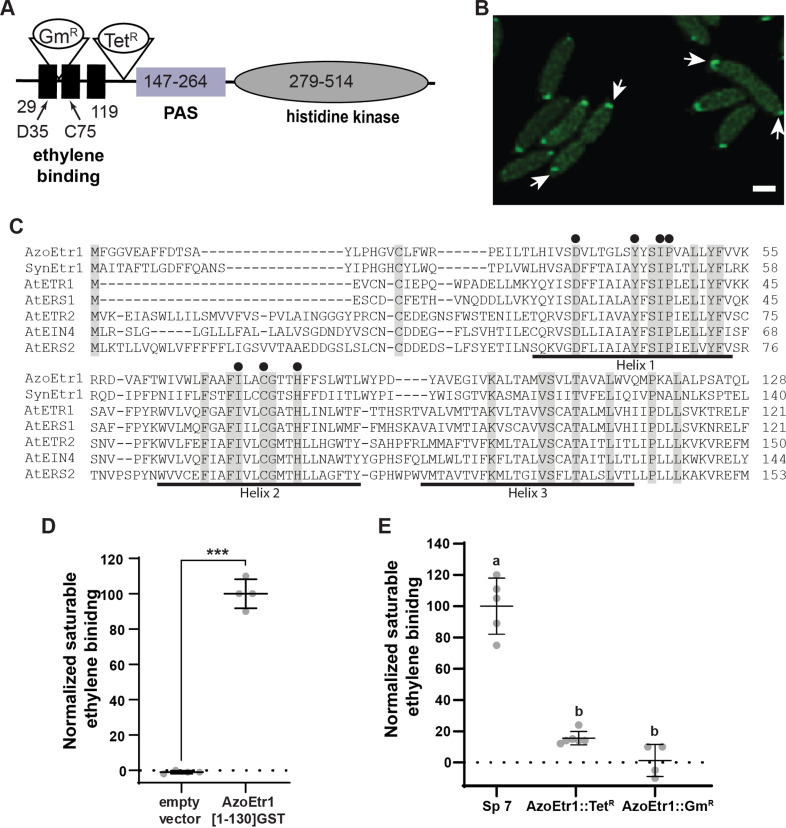
AzoEtr1 binds ethylene. **A)** Predicted domain structure of the AzoEtr1 protein based on sequence homology. Numbers indicate the amino acid range predicted to form each domain. Black rectangles denote predicted transmembrane α-helices that form the ethylene binding domain. Arrows denote the relative location of point mutants constructed in this study. The positions of the gene disruptions using insertion of either a tetracycline (*AzoEtr1::Tet*^*R*^) or a gentamycin (*AzoEtr1::Gm*^*R*^) resistance gene are shown. **B)** Fluorescence confocal microscopy was used to image AzoEtr1-YFP constitutively expressed in *A. brasilense* as described in the materials and methods. Arrows mark focal accumulation of AzoEtr1-YFP at the poles distal to cell division. Scale bar = 1 µm. **C)** Alignment of the AzoEtr1 predicted ethylene binding domain with this domain from the receptor characterized in *Synechocystis* (SynEtr1) and the five receptors from *A. thaliana* (AtETR1, AtERS1, AtETR2, AtEIN4, AtERS2). The seven amino acids that correspond to the residues required for binding of ethylene to AtETR1 as determined with alanine-scanning mutagenesis [31] are marked with circles. Grey highlights identical amino acids. The approximate locations of the three transmembrane helices are marked based on Wang et al (2006) [31]. **D)** Ethylene-binding activity to equal amounts of yeast expressing the binding domain of AzoEtr1 fused to GST (AzoEtr1(1-130)GST) or empty vector. Data were normalized to binding activity in AzoEtr1(1-130)GST-expressing yeast. *** *p* value ≤ 0.001 compared to empty vector as determined by Student’s t-test. **E)** Ethylene-binding activity to equal amounts of *A. brasilense* comparing wild-type (Sp7), AzoEtr1:Tet^R^, and AzoEtr1::Gm^R^. Data were normalized to ethylene-binding activity in wild-type. Different letters denote statistical differences as determined using ANOVA with a *p* value ≤ 0.05. D,E- Saturable ethylene binding was calculated by subtracting the amount of ethylene bound in the presence of excess ^12^C-ethylene (non-specific binding) from the amount of ethylene bound in the absence of ^12^C-ethylene (total binding) as described in the materials and methods. Data shown represents the average ± SD.

In plants, ethylene perception is mediated by a family of receptors that contain a conserved N-terminal domain formed by three transmembrane α-helices and seven conserved amino acids known to be required for binding to AtETR1 from *A. thaliana* [31,52]. Using the amino acid sequence of this domain from AtETR1, we previously determined that many bacterial species contain genes that encode proteins predicted to contain a functional ethylene-binding domain [27,32]. The genome of *A. brasilense* Sp7 contains one such gene (locus tag OH82_RS30505) that we call *Azoetr1*. This gene is predicted to encode a protein 525 aa long with an ethylene-binding domain at the N-terminus comprised of three transmembrane α-helices followed by a PAS (Per-Arnt-Sim), and histidine kinase domain at the C-terminus (Figs 1A, S1A). We observed the accumulation of AzoEtr1-YFP at one cell pole when tagged at the C-terminus (Fig 1B). In dividing cells, focal accumulation of AzoEtr1-YFP was observed at poles distal to cell division suggesting that AzoEtr1 is localized at the polar flagellum site. There is a predicted response regulator encoding gene (*Azorr*^*etr1*^) located just downstream of *Azoetr1* in the genome (locus tag OH82_RS30510) that is predicted to encode a protein that is 135 amino acids long and contains no other recognizable output motif (S1B, S2 Figs). Upstream of the *Azoetr1* gene in the genome and in the same orientation are genes predicted to encode a peptide chain release factor 3, an aspartate-semialdehyde dehydrogenase, a maltodextrin or glycogen phosphorylase, and a hypothetical protein (S2 Fig). There is a small overlap in nucleotides between the start codon of *Azorr*^*etr1*^ and the stop codon of *Azoetr1* suggesting that the two genes are co-transcribed, perhaps as part of an operon (S3 Fig). This gene orientation suggests that AzoEtr1 is a receptor for a two-component signaling system.

Alignment of the N-terminal region of AzoEtr1 with the five ethylene receptors from *A. thaliana* and the one receptor from *Synechocystis* showed that AzoEtr1 has 23 amino acids that are identical to the other characterized ethylene receptors (Fig 1C). This includes the seven amino acids necessary for the binding of ethylene to AtETR1 [31]. Other species of *Azosprillum* also contain putative ethylene receptors with these seven conserved amino acids (S4 Fig). We tested whether AzoEtr1 could directly bind ethylene by expressing the coding sequence for the first 130 amino acids of AzoEtr1 fused to glutathione *S*-transferase (AzoEtr1[1-130]GST), in *Pichia pastoris*. Using radioligand binding assays with ^14^C_2_H_4_ we found that cells expressing AzoEtr1[1-130]GST contained saturable ethylene-binding sites (Fig 1D). Control experiments with *P. pastoris* expressing the empty vector (pPICZ) confirmed that this yeast has no detectable saturable ethylene binding activity as shown previously [32,53]. These results demonstrate that the N-terminal portion of AzoEtr1 is capable of directly binding ethylene.

Based on these results, we examined the binding of ethylene to *A. brasilense* Sp7 and found that these cells had saturable ethylene binding sites (Fig 1E). Disruption of AzoEtr1 with either a tetracycline resistance insert (AzoEtr1::Tet^R^) downstream of helix 3 or a gentamicin resistance insert (AzoEtr1::Gm^R^) between helices 1 and 2 (Fig 1A) resulted in cells with little or no saturable ethylene binding (Fig 1E). Thus, *A. brasilense* Sp7 cells bind ethylene and this binding is mediated by AzoEtr1.

### AzoEtr1 is a functional ethylene receptor that affects biofilm formation

**Fig 2:**
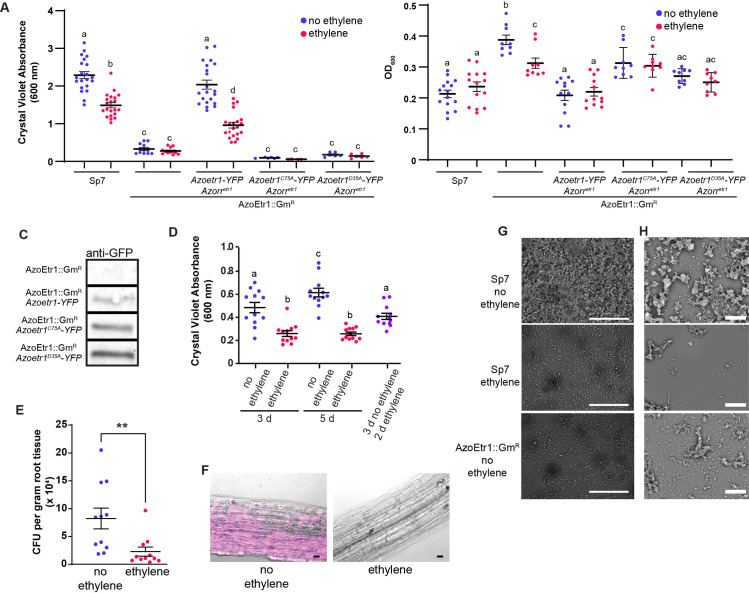
Ethylene reduces biofilm formation and *A. thaliana* root colonization. **A)** Biofilm formation after 3 days in the presence or absence of 100 ppb ethylene was assayed using crystal violet staining. The extent of biofilm formation was compared between wild-type (Sp7) and AzoEtr1::Gm^R^, and AzoEtr1::Gm^R^ transformed with *Azoetr1-YFP* or two point mutants (*Azoetr*^*D35A*^*-YFP*, *Azoetr1*^*C75A*^*-YFP*). All three were co-transformed with a plasmid containing *Azorr*^*etr1*^ using a separate plasmid. **B)** Growth was evaluated by measuring the OD_600_ of the planktonic culture in each assay well in samples treated as in A. **C)** Proteins from membranes of AzoEtr1::Gm^R^ and AzoEtr1::Gm^R^ transformed with either *Azoetr1-YFP*, *Azoetr*^*D35A*^*-YFP*, or *Azoetr1*^*C75A*^*-YFP* were analyzed with immunoblots probed with anti-GST antibodies to confirm the expression of the receptors. **D)** Biofilm formation was allowed to occur for 3 or 5 days in the presence or absence of 100 ppb ethylene compared to 3 days in the absence of ethylene followed by 2 days with ethylene. A, B, D) Data is the average ± SEM. Different letters denote statistical differences as determined using ANOVA with a *p* value ≤ 0.05. **E)** The ability of *A. brasilense* cells to form colony forming units on ethylene-insensitive *ein2-5 A. thaliana* roots was evaluated 24 hours post-inoculation in the absence or presence of 100 ppb ethylene. Data is the average ± SEM. ** *p* value ≤ 0.01 compared to the no ethylene control as determined by Student’s t-test. **F)** Representative fluorescent micrographs of YFP expressing *A. brasilense* on the surface of *ein2-5* roots in the absence or presence of 100 ppb ethylene. Scale bars 10 µm. **G)** Light microscopic images of *A. brasilense* cells grown under biofilm conditions for 1 day showing cell attachment. **H)** SEM images of *A. brasilense* cells grown under biofilm forming conditions for 3 days. G, H) Wild-type cells (Sp7) treated with ethylene-free air or 100 ppb ethylene and AzoEtr1::Gm^R^ cells treated with ethylene-free air are shown.

We wished to determine whether AzoEtr1 is a functional ethylene receptor that initiates changes in cell physiology, behavior, or both. Ethylene has previously been shown to increase biofilm formation in the cyanobacterium *Synechocystis* [32] leading us to examine this trait in *A. brasilense*. To do this we treated *A. brasilense* with 100 ppb ethylene for three days under biofilm conditions. This level of ethylene is commonly used in plant research and has been shown to affect the physiology of two cyanobacteria, *Synechocystis* and *Geitlerinema* sp. PCC 7105, with no obvious adverse effects [32,37,40]. Ethylene reduced biofilm formation by Sp7 and both the AzoEtr1::Gm^R^ and AzoEtr1::Tet^R^ disruptants reduced biofilm formation in either the absence or presence of 100 ppb ethylene (Figs 2A, S5A). The application of ethylene did not measurably alter total cell growth under these conditions (Fig 2B). By contrast, disruption of AzoEtr1 led to increased growth (Figs 2B, S5B). Thus, the reduction in biofilm formation is not a consequence of reduced growth. Disruptant cells also showed alterations in other traits associated with stress such as corrugation of the colony surface, increased sensitivity to H_2_0_2_, and higher accumulation of carotenoids (S6A-D Fig). The application of ethylene to Sp7 cells did not alter the colony surface or H_2_0_2_ sensitivity. Disruption of AzoEtr1 did not affect aerotaxis (S6E Fig).

To confirm that disruption of AzoEtr1 causes a reduction in biofilm formation, we transformed AzoEtr1::Gm^R^ cells with *Azoetr1-YFP*, as well as *Azoetr*^*D35A*^*-YFP*, or *Azoetr1*^*C75A*^*-YFP* mutant genes predicted to not bind ethylene. In AtETR1, C65 is needed to coordinate the copper cofactor required for ethylene binding and aligns with C75 in AzoEtr1 [29]. Computational modeling predicts that the D25 residue in AtETR1 (aligns with D35 in AzoEtr1) is important for positioning H69 (H79 in AzoEtr1) in helix 2 to coordinate the copper cofactor; D25 is also predicted to form a polar bond with a K91 in helix 3 (K98 in AzoEtr1) which is involved in receptor output [54]. Mutation of the C65 or D25 residue eliminates ethylene-binding activity in AtETR1 and mutation of the comparable C residue in SynEtr1 also eliminates binding activity [31,32,52]. AzoEtr1 has characteristics of a two-component receptor which typically signal via a response regulator protein. The presence of a predicted response regulator encoding gene (*Azorr*^*etr1*^) in the genome next to *Azoetr1* suggests that the output of this signaling system is via AzoRR^etr1^. Because we predicted that the AzoEtr1::Gm^R^ disruption would disrupt *Azorr*^*etr1*^, all three of these lines were co-transformed with parental *Azorr*^*etr1*^ using a separate plasmid as detailed in the materials and methods. Transformation with *Azoetr1-YFP* rescued biofilm formation levels in the absence of ethylene and the application of 100 ppb ethylene reduced this similar to what was observed in Sp7 (Fig 2A). Transformation of AzoEtr1::Gm^R^ cells with either *Azoetr*^*D35A*^*-YFP* or *Azoetr1*^*C75A*^*YFP* mutant genes failed to rescue biofilm formation. Transformation with *Azoetr1-YFP* reduced cell growth to wild-type levels, whereas, the mutant receptor transgenes had a smaller effect on cell growth (Fig 2B). It is possible that the ectopic expression of *Azorr*^*etr1*^ on a separate plasmid from *AzoEtr1* could be reducing the relative expression of one or both genes to affect these results. Expression of the transgenic expressed AzoEtr1 receptor proteins was confirmed with immunoblots (Fig 2C) and the relative transcript abundance of *Azorr*^*etr1*^ was similar between the complementation lines (S7 Fig). This supports the conclusion that the mutant receptors are non-functional, although, it does not rule out other confounding factors from using two plasmids for ectopic protein expression. This pattern of rescue is similar to what is observed in comparable mutants in the *Synechocystis* ethylene receptor, SynEtr1, resulting in a non-functional protein [32]. In contrast, this is distinct from results in plants where comparable mutations result in a protein that is functional and signals, but fails to bind ethylene and turn off [52,55]. The transformation of AzoEtr1::Gm^R^ cells with empty vector plasmids did not rescue the biofilm phenotype and had variable effects on total cell growth (S8 Fig).

We tested whether ethylene disperses biofilms by first allowing biofilm formation for three days in ethylene-free air and then applying 100 ppb ethylene for 2 days. This was compared to biofilm formation after 3 or 5 days in the absence of ethylene (Fig 2D). Our results demonstrate that ethylene did not disperse biofilms that had already formed because the amount of biofilm formed was statistically indistinguishable between 3 days with no ethylene and 3 days with no ethylene followed by application of ethylene. However, additional biofilm formation was blocked since more biofilm formed after 5 days with no ethylene compared to 3 days with no ethylene followed by ethylene for 2 days. These results demonstrate that AzoEtr1 is a functional ethylene receptor that affects bacterial traits involved in stress resistance and host colonization, the first to be described in a proteobacterium.

### AzoEtr1 is a functional ethylene receptor that affects root colonization

Ethylene inhibits biofilm formation resulting in reduced cell aggregation. There are many sources of ethylene in soil including plants and bacteria [20]. To determine whether *A. brasilense* Sp7 uses ethylene as a signal to communicate with other *A. brasilense* cells, we measured ethylene production by the bacteria. To do this we grew liquid cultures in sealed flasks overnight, normalized the OD_600_ to 1.0, and incubated the cells with known precursors of ethylene biosynthesis for two hours. We sampled the headspace using a laser-based detector. No ethylene was detected above background levels even in the presence of precursors known to increase ethylene production in various bacteria species and plants [56] (S9 Fig). Thus, *A. brasilense* does not produce ethylene under the conditions tested.

Ethylene could act as a cross-kingdom signal from plants to *A. brasilense* as EPS production and biofilm formation are important for colonization of plant roots by *A. brasilense* [57,58]. This led us to question whether ethylene and AzoEtr1 affect root colonization. To evaluate the influence of ethylene on the ability of bacterial cells to colonize roots, we used *ethylene insensitive 2-5* (*ein2-5*) *A. thaliana* and *never ripe* (*nr*) tomato which do not respond to ethylene [59,60], so that we were only affecting bacteria cells with the application of ethylene. Application of 100 ppb ethylene reduced the ability of *A. brasilense* to colonize the roots of both *ein2-5* and *nr* one day post-inoculation (Figs 2E,F, S10A). Disruption of AzoEtr1 also reduced tomato root surface colonization and fluorescence microscopy showed a loss of cell aggregates on tomato root surfaces (S10B-D Fig).

To investigate the nature of the surface attachment that was reduced by the application of ethylene, we allowed cells to attach to poly-L-lysine-coated slides [61] so that we could visualize the cells under these conditions (Fig 2G). This showed that untreated wild-type cells attached on the slide surface as a dense biomass after 24 h. In contrast, the application of 100 ppb ethylene or disruption of AzoEtr1 resulted in little biomass buildup on the slide and only individual cells or small clusters were visible. To examine for the presence of exopolysaccharide (EPS)-dependent cell aggregation, scanning electron microscopy was performed on samples grown under biofilm conditions (Fig 2H). These images show that wild-type cells in ethylene-free air form large aggregates of cells that contain extracellular fibrils. Both the ethylene-treated and mutant strains did not show large aggregations, and where small aggregates of cells existed, there was little extracellular material visible. Together, these results suggest that ethylene inhibits the EPS production and the aggregation of cells, thereby preventing attachment to surfaces and accumulation of cell biomass.

### *A. brasilense* responds to low levels of ethylene within hours of application

**Fig 3:**
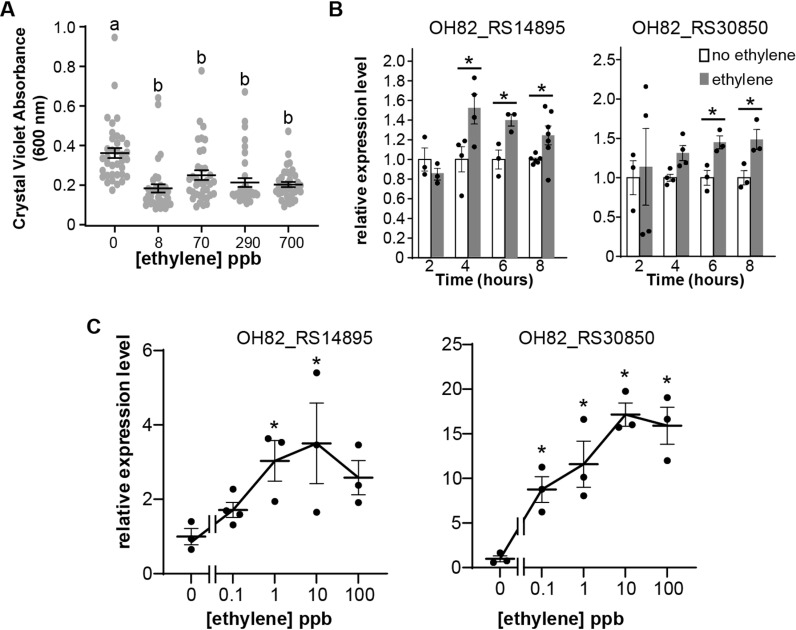
*A. brasilense* responds to low levels of ethylene. **A)** Cells were exposed to different levels of ethylene for 24 hours under biofilm conditions and crystal violet used to measure biofilm formation. Different letters denote statistical differences as determined using ANOVA with a *p* value ≤ 0.05. **B)** Cells were exposed to 100 ppb ethylene for different times and real-time RT-qPCR used to measure the transcript levels of two genes. Data were normalized to housekeeping genes as described in the materials and methods and then to the levels in the ethylene-free controls at that time point. The average ± SEM shown (* *p* value ≤ 0.05 compared to the no ethylene control at that time point as determined by Students t-test). **C)** The gene transcript abundance of these two genes was examined with real-time RT-qPCR as a function of ethylene dose 6 hours after application of the indicated dose of ethylene. Data were normalized to housekeeping genes as noted in the materials and methods and then to the no ethylene control. The average ± SEM shown. * *p* value ≤ 0.05 compared to no ethylene control as determined by ANOVA.

Plants have differential responses over a wide range of ethylene concentrations from over 100 ppm down to 0.2 ppb where transient responses occur within minutes of application [62,63]. We previously showed that the cyanobacteria *Synechocystis* and *Geitlerinema* have physiological responses to the application of ethylene at concentrations as low as 8 ppb (the lowest dose examined), and changes in the transcript levels of specific genes in *Synechocystis* are altered by as low as 1 ppb ethylene [40]. Therefore, we were interested in the sensitivity of *A. brasilense* to ethylene. To examine this, we conducted biofilm assays over a range of ethylene concentrations from 8 to 700 ppb. This revealed that ethylene down to 8 ppb reduced biofilm formation to a similar magnitude as all higher concentrations used (Fig 3A). Thus, the threshold for this change was below 8 ppb, similar to what we reported for the effects of ethylene on the physiology of cyanobacteria [40].

Biofilm assays were conducted over a long period and flow-through chambers were used to deliver ethylene so that O_2_ and CO_2_ were kept constant. However, a limitation of this method is the difficulty in reliably delivering ethylene doses below approximately 8 ppb. We therefore conducted shorter-duration experiments examining changes in the transcripts of two selected genes using much lower ethylene doses where ethylene was simply injected into sealed chambers for several hours. For this we chose OH82_RS30850 (annotated as pyruvate dehydrogenase complex E1 subunit beta) and OH82_RS14895 (annotated as an amino acid permease) because our RNA sequencing (RNA-seq) showed that both of these genes are upregulated by the application of ethylene (see next section). We first determined how quickly gene transcript changes could be measured using real-time RT-qPCR. In *Synechocystis*, ethylene rapidly affects the transcript levels of genes with changes occurring within 30 min [32]. Here we observed no change in transcript levels before four hours after addition of 100 ppb ethylene and OH2_RS30850 did not show a response until six hours after ethylene (Fig 3B). Therefore, we conducted dose-response measurements of these genes 6 h after application of ethylene treatment (Fig 3C). The transcript abundance of OH82_RS30850 increased with ethylene doses as low as 0.1 ppb. A similar trend was observed for OH82_RS14895; however, a statistically significant increase was not observed until 1 ppb. These ethylene levels are below the threshold for most plant responses.

### Ethylene causes wide-spread changes in the metabolism of *A. brasilense*

**Fig 4:**
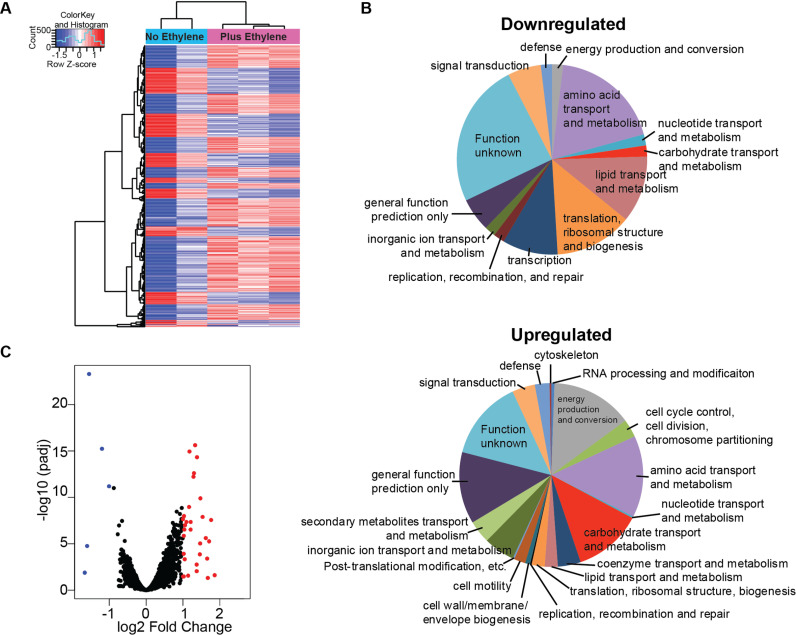
Global gene transcript changes caused by ethylene. *A. brasilense* cells were grown in liquid culture as detailed in the materials and methods for 4 h in the presence or absence of 100 ppb ethylene. Samples were prepared as detailed in the materials and methods and RNA-seq analysis and bioinformatics analyses was carried out by GeneWiz (South Plainfield, NJ, USA). **A)** Heatmap showing relative abundance of each gene in individual control (no ethylene) and ethylene-treated samples. Included are gene transcripts that have a log_2_ change ≥ |0.5| with an adjusted *p*-value ≤ 0.05. **B)** Pie charts showing the percent of genes downregulated (top) or upregulated (bottom) by ethylene in various Gene Ontology categories using the Kyoto Encyclopedia of Genes (KEGG). **C)** Volcano plot showing global transcriptional changes caused by ethylene treatment. Each data point represents a gene. Genes with an adjusted *p*-value ≤ 0.05 and a log2 fold ≥ 1 are indicated by red dots. Genes with an adjusted *p*-value ≤ 0.05 and as log2 fold change ≤ -1 are indicated by blue dots.

To further explore the effects of ethylene on bacterial physiology, we used RNA-seq to examine transcriptomic changes in cells grown in liquid culture with NaNO_3_ as a nitrogen source for 4 h in the presence or absence of 100 ppb ethylene. Principal component analysis (PCA) showed that the ethylene-treated samples clustered separately from the controls (S11A Fig). However, one of the control samples did not cluster with the other two controls; therefore we performed a new PCA analysis without this sample (S11B Fig). Further analyses were conducted without this control sample. Using an adjusted *p* value (Benjamini-Hochberg Procedure) less than 0.05, we observed that 395 of the 6274 genes responded to ethylene with a log_2_ change ≥ |0.5|. Of these, 51 were downregulated and 344 were upregulated (Fig 4A and S1 Data). Gene Ontology analysis showed that many of the annotated genes are predicted to be involved in metabolism including energy production and the metabolism or transport of amino acids, carbohydrates, lipids, secondary metabolites, coenzymes, and inorganic ions (Fig 4B). Of these 395 transcripts, 36 had a log_2_ change ≥ |1.0| with 31 upregulated and five downregulated (Fig 4C and Table 1). When examining the top 36 genes in the context of the entire differentially expressed gene list, many grouped with other genes in the genome that were similarly regulated by ethylene with an adjusted *p* value ≤ 0.05, but with smaller amplitude changes in transcript abundance (S1 Table). These included 15 genes annotated to be involved in transport, 11 potentially involved in redox reactions, and several components of the pyruvate dehydrogenase complex. There were also several other genes that grouped together on the genome and showed significant upregulation, but did not include the top 36 genes (S2 Table). In these groups six genes are annotated as ABC transporters, eight that contain coenzyme A, and six hydratases/dehydratases. Thus, genes in these groups are likely to be co-transcribed and may represent operons. The levels of *Azoetr1* and *Azorr*^*etr1*^ transcripts were not significantly altered by ethylene treatment in the RNA-seq dataset. qPCR of these genes revealed only a small transient change in *Azoetr1* and no change in *Azorr*^*etr1*^ caused by the application of ethylene (S12 Fig).

**Table 1:** Top 36 Gene Transcripts Affected by Ethylene^a^.

Locus	Gene Symbol	Description	Log_2_Fold change	Adjusted *p* value^b^
OH82_RS00065		DUF6455 family protein	1.67	4.87 × 10^-2^
OH82_RS03315		cytochrome c	−1.66	1.32 × 10^-2^
OH82_RS06780		hypothetical protein	1.66	3.97 × 10^-4^
OH82_RS07185		dimethyl sulfoxide reductase anchor subunit	1.47	9.42 × 10^-6^
OH82_RS07200		CoA-binding protein	1.30	2.50 × 10^-13^
OH82_RS07205		acetate-CoA ligase family protein	1.33	2.37 × 10^-16^
OH82_RS07210		aldehyde dehydrogenase family protein	1.71	5.56 × 10^-6^
OH82_RS07215		tripartite tricarboxylate transporter permease	1.37	1.76 × 10^-3^
OH82_RS07365	*pfkB*	1-phosphofructokinase	1.46	1.38 × 10^-4^
OH82_RS07645		Gfo/Idh/MocA family oxidoreductase	1.00	3.42 × 10^-2^
OH82_RS09645		hypothetical protein	1.53	1.29 × 10^-8^
OH82_RS11335	*pspC*	envelope stress response membrane protein PspC	1.14	2.77 × 10^-2^
OH82_RS11435		ABC transporter permease subunit	1.02	1.41 × 10^-6^
OH82_RS12700		polymer-forming cytoskeletal protein	1.29	5.98 × 10^-13^
OH82_RS13415		ammonium transporter	−1.01	6.42 × 10^-12^
OH82_RS13525	*ccmD*	heme exporter protein CcmD	1.86	2.46 × 10^-2^
OH82_RS15330		branched-chain amino acid ABC transporter permease	1.03	1.05 × 10^-8^
OH82_RS16030	*ftsZ*	cell division protein FtsZ	1.17	1.09 × 10^-9^
OH82_RS16645	*exbB*	tonB-system energizer ExbB	1.02	1.22 × 10^-4^
OH82_RS19240		ABC transporter permease subunit	1.05	4.66 × 10^-4^
OH82_RS20060		MerR family DNA-binding transcriptional regulator	−1.60	1.76 × 10^-5^
OH82_RS21855		hypothetical protein	1.02	3.38 × 10^-2^
OH82_RS22905		methyltransferase domain-containing protein	1.04	2.94 × 10^-7^
OH82_RS23775		SCP2 sterol-binding domain-containing protein	−1.20	5.74 × 10^-16^
OH82_RS23780		long-chain fatty acid-CoA ligase	−1.55	4.94 × 10^-24^
OH82_RS24150		cobalamin biosynthesis protein	1.21	2.98 × 10^-7^
OH82_RS25885		3-oxoacyl-ACP reductase FabG2	1.62	2.45 × 10^-6^
OH82_RS27590		CoA ester lyase	1.37	9.12 × 10^-3^
OH82_RS28175		YeeE/YedE family protein	1.21	4.54 × 10^-8^
OH82_RS28815		acetoin dehydrogenase dihydrolipoyllysine-residue acetyltransferase subunit	1.47	1.28 × 10^-10^
OH82_RS29790		2-hydroxyacid dehydrogenase	1.09	4.54 × 10^-8^
OH82_RS29960		SPOR domain-containing protein	1.00	2.24 × 10^-8^
OH82_RS30845		pyruvate dehydrogenase complex dihydrolipoamide acetyltransferase	1.38	4.69 × 10^-15^
OH82_RS30850		pyruvate dehydrogenase complex E1 component subunit β	1.18	1.13 × 10^-15^
OH82_RS31100		hypothetical protein	1.76	2.78 × 10^-8^
OH82_RS32115		tripartite tricarboxylate transporter TctB family protein	1.06	1.02 × 10^-7^

^a^Cells were treated with 100 ppb ethylene as detailed in the materials and methods. RNA-seq analysis was conducted and genes that had an adjusted *p* value ≤ 0.05 and changed expression levels |Log_2_fold ≥1| were selected.

^b^Benjamini-Hochberg Procedure used.

**Fig 5:**
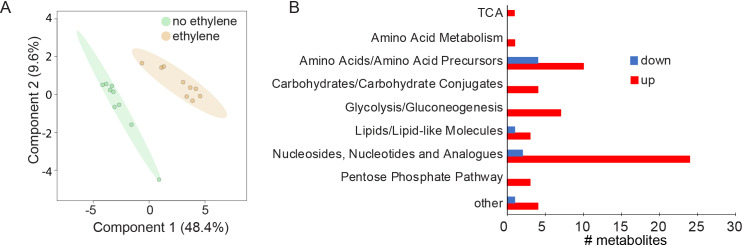
Ethylene alters the levels of many metabolites. *A. brasilense* cells were treated with 100 ppb ethylene or ethylene-free air for 24 h. At this time, untargeted metabolomics analysis was conducted as described in the Materials and Methods. **A)** Partial least squares discriminant analysis of data. **B)** Pathway analysis using the Kyoto Encyclopedia of Genes (KEGG) showing pathways of the 69 differentially accumulated metabolites.

RNA-seq analyses predicted that ethylene treatment elicited major metabolic changes in the bacteria. Therefore, we performed untargeted metabolomics to assess the patterns of metabolites in cells treated with ethylene versus untreated cells under the same conditions as used for the RNA-seq experiment. Cells treated with ethylene had significantly altered metabolite profiles between treated and untreated cells as determined by partial least square discriminant analysis (Fig 5A). Of the 123 metabolites detected, 69 were significantly altered (*p* ≤ 0.1) between the two conditions with 60 enriched and nine depleted (S3 Table). Many metabolites in carbohydrate and carbon metabolism such as 3-phosphoglycerate, glyceraldehyde 3-phosphate, fructose 1,6-bisphosphate, glucose phosphate, phosphoenolpyruvate, pyruvate, and UDP-glucose were increased by ethylene treatment. A key regulator of nitrogen metabolism, 2-oxoglutarate, was also increased [64,65]. By contrast, the second messenger cyclic di-GMP, which is involved in the regulation of biofilm formation and metabolism [66,67], was reduced by ethylene. Pathway analysis showed that nucleotides and analogs were the most highly altered pathways, followed by amino acid metabolism (Fig 5B). These observations suggest that nitrogen metabolism is a major target of ethylene signaling in this bacterium.

### Ethylene mediates changes in the balance between carbon and nitrogen metabolism

Our transcriptomic data indicated that carbon metabolism was upregulated and nitrogen metabolism was downregulated by ethylene. Non-targeted metabolomics also indicated that ethylene treatment increased the abundance of metabolites related to glycolysis and gluconeogenesis. These observations suggest that the perception of ethylene can alter the balance between carbon and nitrogen metabolisms in *A. brasilense*.

Three genes with regulatory roles in nitrogen metabolism were downregulated by ethylene in the RNA-seq datasets: *glnA* (OH82_RS05785) encoding a glutamine synthetase, *glnB* (OH82_RS05780) encoding a PII regulatory protein, and *amtB* (OH82_RS13415) encoding an ammonium transporter. All three gene products have been shown to regulate *GlnAB* and play important roles in the regulation of nitrogen metabolism in *A. brasilense* [68–73] as well as many other bacteria. GlnB is a PII protein implicated in regulating bacterial metabolism in response to changes in the cellular carbon to nitrogen ratio [68,70,72,73] and is a major regulator of nitrogen metabolism including free-living nitrogen fixation. *A. brasilense* also possesses a second PII protein, GlnZ, that plays a distinct role from that of GlnB in the regulation of nitrogen fixation [72,70,74–75]. The *glnZ* gene was not altered by ethylene in our RNA-seq data.

**Fig 6:**
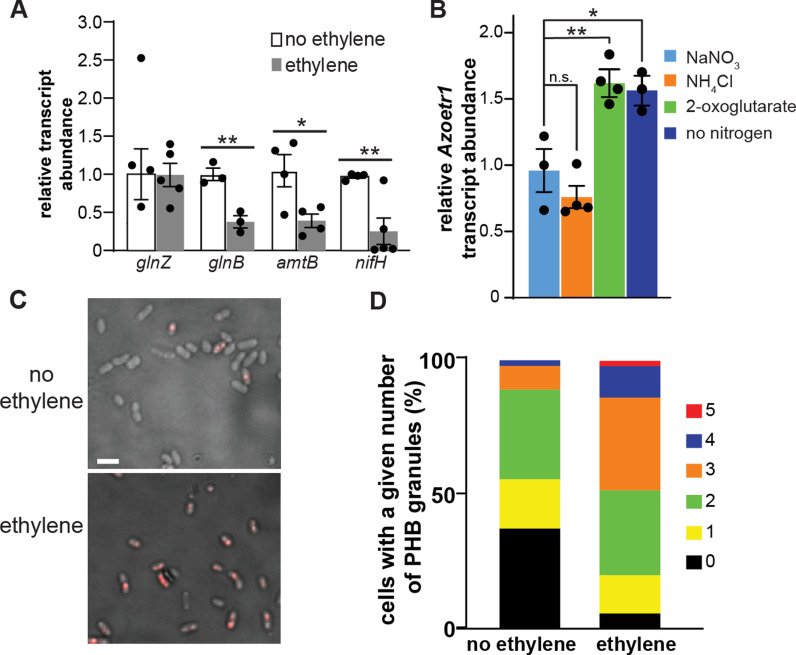
Ethylene alters the metabolic balance and stimulates poly-hydroxybutyrate (PHB) accumulation. **A)** Cells were grown with no nitrogen source in the presence and absence of 100 ppb ethylene for 24 hours at which time samples were processed and real-time RT-qPCR used to measure the transcript levels of the indicated genes. Data were normalized to expression of housekeeping genes and then to expression in the absence of ethylene as described in the materials and methods. Data is the average ± SEM. * *p* value < 0.05; ** *p* value < 0.01 compared to the no ethylene control as determined by Student’s t-test. **B)** Cells were grown overnight with 10 mM of the indicated nitrogen source or no added nitrogen with no shaking. At this time RNA was extracted and real-time RT-qPCR used to measure the transcript level of *Azoetr1*. Data was normalized to levels in the presence of NaNO_3_ and housekeeping genes as described in the materials and methods. Data is the average ± SEM. Statistical difference from NaNO_3_ was determined with ANOVA. n.s. not significant; * *p* value < 0.05; ** *p* value < 0.01. **C, D)** Cells were grown in MMAB in the presence and absence of 100 ppb ethylene for 24 hours at which time the cells were stained with Nile red. C) Representative DIC merged with fluorescent images of cells in control cells treated with 100 ppb ethylene. Scale bar = 1 µM. D) Stack graph showing the number of PHB granules accumulated per cell in control (n = 389) and ethylene-treated cells (n = 251).

To validate the changes in the abundance of *glnB* and *amtB* as a result of ethylene treatment, we used real-time RT-qPCR to compare the expression of *glnB* and *amtB* under nitrogen fixing conditions and included *glnZ* as a negative control. Consistent with the RNA-seq data, we found that the application of 100 ppb ethylene reduced the expression of both *glnB* and *amtB* but had no effect on *glnZ* (Fig 6A). The promoter region of *Azoetr1* contains a putative binding site for the sigma-54 factor RpoN (S3 Fig). RpoN controls many nitrogen-related behaviors in *A. brasilense* including nitrogenase expression [76]. Given the regulatory role of GlnB in the induction of nitrogen fixation and the role of RpoN in controlling nitrogen fixation gene expression, we tested the effect of ethylene treatment on the induction of *nifH* (OH82_RS22550) that encodes a subunit of the nitrogenase enzyme [77–79]. As expected, the transcript levels of *nifH* were reduced in the presence of 100 ppb ethylene (Fig 6A). *Azoetr1* expression was similar when grown in the presence of either NaNO_3_ or NH_4_Cl as the nitrogen sources (Fig 6B). Consistent with ethylene signaling being linked to nitrogen metabolism, *Azoetr1* expression was increased either in the absence of a nitrogen source (nitrogen fixation conditions) or in the presence of 2-oxoglutarate which mimics the high carbon to nitrogen ratio signal that regulates GlnB activity. Together these findings are consistent with ethylene mediating changes in the balance between carbon and nitrogen metabolism, most likely by affecting the abundance of *glnB* transcript levels, thus altering the ability of cells to respond to shifts in the carbon-to-nitrogen ratio.

An imbalance between nitrogen and carbon metabolism regulates the accumulation of poly-hydroxybutyrate (PHB) which is also regulated by GlnB in *A. brasilense* [80]. PHB serves as a carbon reserve for many bacteria and its accumulation in *A. brasilense* promotes stress endurance [81]. Using cells stained with the lipophilic dye Nile Red and microscopy, we showed that ethylene treatment increased the number of cells that contained PHB granules, including a statistically significant increase (Mann–Whitney U test *p* < 0.001) in the median number of PHB granules per cell from 1 to 3 under these conditions (Fig 6C, D and S2 Data). These results are consistent with the RNA-seq data showing a decrease in the expression of a gene encoding a poly-hydroxybutyrate depolymerase, *phaZ*, (OH82_RS11420), which is involved in the breakdown of PHB [82].

## Discussion

Plants respond to environmental challenges by recruiting different subpopulations of beneficial, non-symbiotic bacteria in the rhizosphere, but little is known about how this selection occurs [4,83]. The observation that plants can select some of their rhizospheric bacterial partners implies an exchange of molecular signals to select the most beneficial microorganisms from a diverse pool of candidates in the rhizosphere. Ethylene levels in the soil can reach sufficiently high concentrations to elicit responses in plants and it is clear that bacteria modulate ethylene responses in plants [21,44]. This coupled with the presence of an ethylene receptor in *A. brasilense* led us to hypothesize that root-derived ethylene affects the recruitment of specific rhizosphere bacteria to the root surface. Although ethylene receptors and signal transduction have been well-studied in plants, they have not been extensively studied in non-plant species [24,25,27]. Here, we show that AzoEtr1 directly binds ethylene and the application of ethylene regulates *A. brasilense* physiology including reducing biofilm formation and root colonization. Additionally, the application of ethylene causes wide-spread changes in metabolism that appear to upregulate carbon metabolism and downregulate nitrogen metabolism. Nitrogen metabolism and nutrient availability, including the carbon-to-nitrogen ratio, regulate cell-to-cell aggregation, root attachment, and biofilm formation in *A. brasilense* [84–87]. These data indicate that the perception of ethylene by soil and rhizosphere bacteria, such as *A. brasilense*, regulates root colonization.

Plants contain multiple ethylene receptor isoforms and deleting ethylene receptors causes constitutive ethylene responses with the severity increasing depending on the number of isoforms removed [88,89]. This and other data led to an inverse-agonist model for ethylene receptor signaling where in the absence of ethylene, the receptors are inhibiting down-stream signaling; the binding of ethylene inhibits the receptors leading to activation of down-stream signaling [88,90]. This model also seems to apply to *A. brasilense* (this study) and *Synechocystis* [32] because disrupting the receptor in either bacterium causes constitutive ethylene responses. This is interesting because the signaling pathways downstream of the ethylene receptors are different in each system. In plants, the ethylene receptors predominantly signal via a his-kinase-independent mechanism to a ser/thr protein kinase which functions as a negative regulator of the pathway [91–94]. By contrast, SynEtr1 signals via his autophosphorylation and phosphotransfer to a downstream response regulator, slr1213, which activates transcription of a small non-coding RNA, *csiR1* [36]. Ethylene inhibits his kinase activity of AtETR1 and there is indirect evidence that it also inhibits his kinase activity in SynEtr1 [32,95]. In *A. brasilense*, we predict that signaling from AzoEtr1 involves his autophosphorylation and phosphotransfer to AzoRR^Etr1^, and, based on studies of other receptors we predict that ethylene inhibits AzoEtr1 his autophosphorylation. Deleting the single ethylene receptor isoform in either *A. brasilense* or *Synechocystis* resulted in more extreme phenotypes than the application of ethylene. This is similar to what occurs in plants in which multiple isoforms were deleted and has contributed to the idea that there may be feedback inhibition on the receptors to desensitize the plant to ethylene [24,89,96]. Ethylene receptors in plants also have ethylene-independent roles [97] that might contribute to the more extreme phenotypes. Thus, the bacterial ethylene receptors may have roles in addition to mediating responses to ethylene or there may be feedback mechanisms on the receptors that reduce responses to ethylene as observed in plants [24,25]. Lower nitrogen availability increases *AzoEtr1* transcript abundance which would decrease ethylene sensitivity. *AzoEtr1* contains a putative binding site for the sigma-54 factor RpoN which might mediate this response to nitrogen. Thus, one possible feedback loop may involve RpoN.

The effects of ethylene on *A. brasilense* and *Synechocystis* are not the same. Physiologically, ethylene causes opposite effects on biofilm formation, with an increase in *Synechocystis* [32] and a decrease in *A. brasilense.* Additionally, *Synechocystis* responds quickly (within 30 min) to the application of ethylene [38], whereas *A. brasilense* requires several hours to respond. Deleting SynEtr1 results in faster twitching motility of *Synechocystis* [32], whereas, deletion of AzoEtr1 has no effect on *A. brasilense* swimming motility, since the timing of aerotaxis band formation was unaffected. *Synechocystis* motility is mediated by the coordination of pili and EPS production [98–100]. In contrast, motility in *A. brasilense* relies on a single polar flagellum that powers the movement of individual cells, whereas EPS production is required for biofilm formation and attachment [87,101,102]. Thus, it is not unexpected that the impact of ethylene perception on bacterial behavior differs between these two organisms One point of similarity is that both organisms are very sensitive to ethylene with responses occurring as low as 1 ppb ethylene [40], which is below the threshold for most responses in plants [93].

The ethylene-induced shift that increases carbon metabolism and reduces nitrogen assimilation (nitrogen-replete conditions) and nitrogen fixation (no organic nitrogen present) is intriguing. *A. brasilense* cells adapt their physiology to respond to changes in the availability of carbon relative to nitrogen in their environment by regulating attachment to roots, flocculation, and biofilm formation [84–86]. These traits have been linked to stress endurance and root surface colonization [87,101]. Based on our data, there are several mechanisms to consider by which ethylene may cause the shift in metabolism. Limiting the availability of nitrogen causes the accumulation of 2-oxoglutarate and a concomitant reduction in glutamine levels, which triggers differential signaling by PII proteins [64,65]. The measurements of these resources allow PII proteins, such as GlnB, to function at the center of the regulation of the balance between cellular carbon and nitrogen metabolism by interacting with many unrelated molecular targets to mediate cellular adaptation [68–73,77,103–106]. Our metabolomics data showed that ethylene increases 2-oxoglutarate levels. Given the central role of 2-oxoglutarate in signaling to the PII proteins [64,65], this represents a possible mechanism by which ethylene shifts metabolism by making the bacteria partially “blind” to shifts in nitrogen availability leading to alterations in carbon and nitrogen metabolic processes. This is reflected in the upregulation of carbon metabolism genes such as the 1-phosphofrutokinase *pfkB* and several genes encoding for components of the pyruvate dehydrogenase complex and downregulation of *glnB* which regulates the expression of other nitrogen assimilation (*amtB*) and nitrogen fixation (*nif*H) genes [70]. This effect of ethylene on *glnB* abundance could directly reduce the ability of cells to respond to shifts in carbon-to-nitrogen ratios. The changes in both 2-oxoglutarate and *glnB* are likely to be additive in altering the cells ability to sense changes in nitrogen. Our metabolomics data showed that ethylene also caused a reduction in cyclic di-GMP levels. Given that cyclic di-GMP affects both biofilm formation and metabolism [66,67], ethylene may also be affecting the cells by modulating the levels of this second messenger. At this point, the signaling pathway linking AzoEtr1 to these responses is unknown and it is unclear which of these or combination of these changes are central to responses to ethylene. Characterizing the signal transduction target(s) of AzoEtr1 should help decipher among these possibilities.

The advantages of perceiving ethylene in the rhizosphere are not yet clear. Unlike plants and some bacteria, our results indicated that *A. brasilense* Sp7 cannot biosynthesize ethylene under the conditions tested. This does not rule out that there are certain conditions where *A. brasilense* biosynthesizes ethylene. But, if true that *A. brasilense* does not biosynthesize ethylene, then ethylene must be encountered from external sources. Ethylene levels in the soil are influenced by various factors, including compaction and water saturation and can range from trace amounts to above 10 ppm, which is well above the levels needed to elicit biological responses in plants [20–23]. Our results suggest several possible ecophysiological roles for ethylene in *A. brasilense*. One major source of ethylene in the soil are plant roots, raising the possibility that plant-derived ethylene regulates root colonization by *A. brasilense*. In this role it might be regulating the timing, spatial distribution, or both of colonization of the root surface. Ethylene does not seem to cause cells to leave a biofilm, but can prevent additional cells from becoming part of a biofilm. Colonization of plant roots can lead to increased ethylene biosynthesis by the plant [16,43,107]. Thus, another role of plant-derived ethylene may be to act as a cue for *A. brasilense* to return to a motile life-style, perhaps to avoid competition by other bacteria on the root surface. It is also possible that ethylene is not a plant-derived signal, but is a signal from other microorganisms in the rhizosphere. In this role, ethylene might signal a highly competitive environment, thus signaling *A. brasilense* to remain a free-living cell and store carbon in the form of PHB for when an environment favorable for attachment occurs. However, these roles are not mutually exclusive. Many soil proteobacteria that form associations with plants also contain putative ethylene receptors [27,32,41] suggesting that ethylene acts as a gaseous signal that affects colonization by many species of bacteria.

## Materials and methods

Seeds of tomato var. Floridade were obtained from Zellajake Farm and Garden, var. Microtom from Urban Farmer Seeds & Supplies, and *nr* var. Ailsa Craig were from Gloria Muday. *Arabidopsis* seeds are lab stocks.

### Bacterial strains and growth conditions

Wild-type *Azospirillum brasilense* Sp7 and AzoEtr1 mutants in the same background were used throughout this study. Bacterial strains and plasmids used in this study are listed in Table 2. Cells were grown at 28°C with shaking in either tryptone-yeast (TY) or Minimal Media for *A. brasilense* (MMAB) supplemented with 10 mM NaNO_3_ unless otherwise indicated. For cultures grown in the absence of nitrogen or 2-oxoglutarate, an overnight culture grown in TY was resuspended in the designated media and adjusted to an OD_600_ of 1.0. These cultures were grown overnight without shaking to allow for biological nitrogen fixation. Growth in TY was supplemented with appropriate antibiotic selections (ampicillin 200 µg ml^-1^ for all *A. brasilense*, 10 µg ml^-1^ tetracycline for Tet^R^ insertions, and 50 µg ml^-1^ gentamycin for Gm^R^ insertions).

**Table 2:** List of strains and plasmids used in this study.

Strain/Plasmid	Relevant Characteristics	Reference or Source
A*. brasilense* Sp7	Amp^R^	[102]
*azoEtr1*::Tet^R^	Insertional disruption of *azoEtr1*, Amp^R^, Tet^R^	This work
*azoEtr1*::Gm^R^	Insertional disruption of *azoEtr1*, Amp^R^, Gm^R^	This work
pRH005	Gateway-based destination vector expressing proteins fused with YFP at their C-terminus; Km^R^, Cm^R^	[108]
pBBR1-MCS3	Plac promoter, Tet^R^	[109]
A*. brasilense Sp7* *pRH005-pKan*^*R*^*-YFP*	Sp7 expressing plasmid based YFP under Kan^R^ promoter for constitutive expression.	This work
*azoEtr1*::Tet^R^ pRH005-pKan^R^-YFP	*azoEtr1*::Tet^R^ expressing plasmid based YFP under Kan^R^ promoter for constitutive expression.	This work
A*. brasilense* Sp7 pRH005-Etr1	Sp7 expressing C-terminal Etr1-YFP fusion from pRH005, Amp^R^, Kan^R^	This work
*azoEtr1*::Gm^R^ pRH005-Etr1 pBBR1-MCS3 azoRR^Etr1^	*azoEtr1*::Gm^R^ expressing Etr1-YFP fusion from pRH005 and azoRR^Etr1^ from pBBR1-MCS3, Gm^R^, Kan^R^, Amp^R^, Tet^R^	This work
*azoEtr1*::Gm^R^ pRH005-D35A pBBR1-MCS3 azoRR^Etr1^	*azoEtr1*::Gm^R^ expressing D35A mutant Etr1-YFP fusion from pRH005 and azoRR^Etr1^ from pBBR1-MCS3, Gm^R^, Kan^R^, Amp^R^, Tet^R^	This work
*azoEtr1*::Gm^R^ pRH005-C75A pBBR1-MCS3 azoRR^Etr1^	*azoEtr1*::Gm^R^ expressing C75A mutant Etr1-YFP fusion from pRH005 and azoRR^Etr1^ from pBBR1-MCS3, Gm^R^, Kan^R^, Amp^R^, Tet^R^	This work
*azoEtr1*::Gm^R^ pRH005	*azoEtr1*::Gm^R^ harboring pRH005	This work
*azoEtr1*::Gm^R^ pBBr1-MCS3	*azoEtr1*::Gm^R^ harboring pBBR1-MCS3, Tet^R^	This work
DH5α	RecA1^-^ EndA1^-^ blue/white	Thermofisher
pRK 2013	ColE1 replicon, Tra, KanR Helper plasmid for triparental conjugation	[110]

### Localization of AzoEtr1

Gateway cloning, according to the manufacturer’s recommendations, was used to create C-terminal YFP fusions of AzoEtr1 using the pRH005 destination vector. Gene specific primers were prepared and Sp7 genomic DNA was used as a template. PCR products were then subjected to BP and LR cloning steps to move the PCR product first into pDONR2.1, an expression vector before being moved into pRH005, the destination vector, yielding the YFP fusion constructs under constitutive expression. These constructs were conjugated to wild-type *A. brasilense Sp7* cells. Cells expressing AzoEtr1-YFP were grown and image stacks were captured using a Leica

SP8 confocal microscope. Images were analyzed by the maximum intensity projection of 25 stacks. Images were processed using Lightning deconvolution software.

### Disruption of *Azoetr1* and complementation in *A. brasilense*

*Azoetr1* was disrupted with either a tetracycline resistance insert (AzoEtr1::Tet^R^) downstream of helix 3 or a gentamicin resistance insert (AzoEtr1::Gm^R^) between transmembrane helices 1 and 2. Insertional mutagenesis was performed as previously described [111]. Gateway cloning according to manufacturer recommendations was used to create the C-terminal YFP fusion of AzoEtr1 using the pRH005 destination vector using the 500 bp upstream of *Azoetr1* as the promoter. Gene specific primers were made and Sp7 genomic DNA was used as a template. PCR products then underwent BP and LR cloning steps to move the PCR product first into pDONR2.1, an expression vector before being moved into pRH005, the destination vector, yielding the YFP fusion constructs. To create AzoEtr1^D35A^ and AzoEtr1^C75A^ mutants containing the D35A and C75A point mutations respectively, *Azoetr1* was placed into the pUC19 plasmid and site-directed mutagenesis was performed using the primers listed in S4 Table. After mutagenesis, the same primers used for wild-type *Azoetr1* gateway cloning were again used to move AzoEtr1^D35A^ and AzoEtr1^C75A^ into the pRH005 vector for expression. DH5α *E. coli* were transformed with constructs confirmed by sequencing and mated into AzoEtr1::Gm^R^ cells using the triparental conjugation protocol for *A. brasilense* [111]. Complementation with *Azorr*^*etr1*^ was performed by amplification with gene specific primers encoding restriction cut sites. After digestion of both the template DNA and pBBR1-MCS3 plasmid, *Azorr*^*etr*1^ was ligated into the pBBR1-MCS3 plasmid putting Azorr^Etr1^ expression under the *lac* promoter. pBBR1-MCS3 containing *Azorr*^*etr1*^was mated into AzoEtr1::Gm^R^ pRH005-Etr1, AzoEtr1::Gm^R^ pRH005-Etr1^D35A^, and AzoEtr1::Gm^R^ pRH005-Etr1^C75A^, using a triparental conjugation protocol. No induction of Azorr^Etr1^ with IPTG was necessary because leaky expression was enough to give comparable levels of *Azorr*^*etr1*^ as wild-type *A. brasilense* (S7 Fig).

### Cloning of *Azoetr1* and expression of AzoEtr1 in *P. pastoris*

When we started this project, *A. brasilense* Sp7 had not yet been sequenced. Therefore, we cloned *AzoEtr1* from *A. brasilense* Sp245 (now known as *A. baldaniorum*) [112] and PCR amplified a truncated version (the first 390 bp) of the gene corresponding to the coding region for the putative ethylene-binding domain (first 130 amino acids). Amplification was carried out by PCR with a 5’ *Eco*R1 restriction site and a 3’*Xho*I restriction site using 5’-TGAATTCATGTTCGGTGGCGTGGAAGCCTTC-3’ and 5’-GTCTCGAGGTCGGCGAGTTGCGTGGC**-**3’ primers. Sequencing *A*. *brasilense* Sp7 showed that the amino acid sequences of *A. brasilense* Sp7 and *A. baldaniorum* are identical for the first 130 amino acids used for ethylene-binding assays (S4 Fig). The PCR fragment was subcloned into a pGEM-T vector and DH5α *E. coli* cells transformed with this plasmid. The plasmids were isolated and *AzoEtr1* excised, and subsequently ligated into the *P. pastoris* expression vector, pPIKZA containing a GST tag at the 3’ end of the gene, as previously described [32]. *P. pastoris* was transformed with these plasmids as previously described by [53]. AzoEtr1[1-130]GST was expressed in yeast cells using the protocol described in the Invitrogen *P. pastoris* manual. Following at 48-hour induction with methanol, cells were harvested, washed once with water, frozen in liquid nitrogen, and stored at -80°C until use.

### Whole-cell ethylene-binding assays

^14^C_2_H_4_ was obtained from ViTrax Radiochemicals (Placentia, CA, USA) and trapped in mercury perchlorate as previously described [52,113]. For both bacteria and yeast, cells were thawed and whole-cell ethylene-binding assays were carried out as previously described [29,52,113]. For yeast cells, 1 g fresh weight per sample was used and for bacteria cells, 3 g fresh weight per sample were used. The cells were exposed to 400 ppb ^14^C_2_H_4_ in the presence or absence of excess ^12^C_2_H_4_. Saturable ethylene binding was calculated by subtracting the amount of ethylene bound in the presence of excess ^12^C_2_H_4_ (non-specific binding) from the amount of ethylene bound in the absence of ^12^C_2_H_4_ (total binding). All experiments were repeated at least three times. Yeast data was normalized to saturable binding in cells expressing AzoEtr1, and bacterial cells to saturable binding in *A. brasilense* Sp7.

### Sequence alignment and domain prediction

Sequence alignment was performed using Clustal Omega software (https://www.ebi.ac.uk/jdispatcher/msa/clustalo). Genomic predictions of *Azoetr1* and *Azorr*^*etr1*^ were performed using the Basic Local Alignment Search Tool (https://blast.ncbi.nlm.nih.gov/Blast.cgi). Protein domain predictions were done using Simple Modular Architecture Research Tool (http://smart.embl-heidelberg.de/) [114,115].

### Crystal violet biofilm staining

Biofilm formation was assayed by crystal violet staining [84] in 12-well PVLC plates with the lid pierced above each well with a 27-gauge needle and covered with micropore surgical tape to ensure equal gas flow between the wells. The plates were placed in airtight chambers and treated with the specified concentration of ethylene for 3 days before crystal violet staining. After 3-days, liquid was removed from the wells and stained with 0.1% crystal violet for 20 minutes. Crystal violet was then removed and each sample washed with distilled water three times. After washing, the crystal violet was solubilized in 95% ethanol and the absorbance at 600nm (A_600_) was measured. Growth of cells in separate samples was determined by measuring the OD_600_ of the planktonic culture in each well. Data represent the analysis of at least three biological replicates for each condition which were each analyzed with three technical replicates

### Abiotic surface attachment on slide assays

A*. brasilense* cultures were grown in TY media overnight with appropriate antibiotics and adjusted to an OD_600_ of 1.0 in 1mM NaNO_3_ supplemented MMAB used in previous biofilm experiments. Microscope slides were coated with poly-L-lysine to increase cell adhesion [61] prior to the application of 10 µL samples. These were placed into 500 mL mason jars without cover slips and sealed with lids fitted with rubber septa to inject ethylene. Humidity was maintained in the chambers with wet paper. Samples were treated with either 100 ppb ethylene or ethylene-free air and incubated at 28°C for 24 h. After this time, the slides were removed and imaged on a Zeiss Axio Observer Z1 at 100x.

### Scanning electron microscopy

*A. brasilense* was grown in liquid MMAB media with antibiotics and incubated with shaking at 28°C until the culture reached OD_600_ of 0.4. The samples were centrifuged and washed with Chemotaxis (Che) buffer (10 mM phosphate buffer plus 0.1 mM EDTA) three times, resuspended in biofilm media, and placed in Petri dishes. Silicon chip specimen supports (TED PELLA, Inc.) were sterilized with 95% (v/v) ethanol, coated with poly-L-lysine to increase cell adhesion [61], placed in Petri dishes with cells. The samples were placed in an airtight container and sealed with lids fitted with rubber septa to inject ethylene. Samples were treated with 100 ppb ethylene or ethylene-free air and incubated at 28°C without shaking for 72 h to allow formation of biofilm. The samples were prepared for scanning electron microscopy and visualized using a Hitachi TM3030 scanning electron microscope.

### Root colonization assays

Root colonization assays were performed on tomato and *A. thaliana* with a modification of a prior study [116]. Briefly, tomato seeds were surface sterilized with 50% (v/v) bleach for 30 min to remove and kill microbes on the seed coat, washed three times with distilled H_2_O, plated on 0.8% (w/v) agar plates containing ¼ strength Murashige and Skoog salts [117] and grown for 3d in a 16 h:8 h light:dark cycle at 28°C. Seedlings were then transferred to 50 mL chambers (2 seedlings per chamber) containing Fahraeus media with 0.6% (w/v) agar and inoculated with *A. brasilense*. Four chambers were used for each assay. Colonization assays were performed in the presence or absence of 100 ppb ethylene for 24 hours. The roots were then removed and gently rinsed with distilled water followed by root tissue homogenization using a bead homogenizer. The resulting mixture was serially diluted to 10^-8^ and plated to count the CFUs. The colonization index was calculated as previously described [116], and normalized to either wild-type or ethylene-free conditions.

For *A. thaliana* colonization, seeds were surface sterilized with 70% (v/v) ethanol and 10% (v/v) bleach plus 0.1% (v/v) triton X-100. They were then stratified for 3 days and plated on 0.8% (w/v) agar plates containing ¼ strength Murashige and Skoog salts [117] and grown for 4d in light at 22°C. The seedlings were dipped into concentrated bacteria placed on fresh agar plates. Colonization was allowed to occur for 24 h in the presence or absence of 100 ppb ethylene. Whole seedlings were weighed and homogenized before serial dilution and plating for CFU counting. CFUs per gram of plant fresh weight were calculated.

### Fluorescent microscopy of root colonization

In some cases, root colonization was performed as described above using YFP-expressing *A. brasilense*. These were generated by conjugation with the gateway destination vector pRH005 to express KanR promoter-YFP fusion. For tomato colonization, assays were carried out in microscopy slide-in-chambers containing one tomato plant as previously described [118]. These chambers were maintained at 28°C for 24 h before imaging using a Leica SP8 confocal microscope. The colonization of *A. thaliana* roots was performed as described above. After 24 h, the plants were removed from the wells and placed onto slides with 5 µL of water. Images were taken on a Zeiss Axio Observer Z1 microscope.

### Ethylene measurements

To measure ethylene production by *A. brasilense*, liquid cultures were grown to an OD_600_ of 1.0 and sealed for 2 h before sampling the headspace using an ETD-300 photoacoustic ethylene detector (Sensor Sense B.V., Nijmegen, The Netherlands). Some samples were supplemented with 1 µM 1-aminocyclopropane-1-carboxylate, 1 mM methionine, or 1 mM 2-(methylthio)ethanol as these are known precursors for ethylene biosynthesis in plants and bacteria [56]. The experiments were replicated twice.

### RNA extraction and qRT-PCR

For gene expression analysis, RNA was extracted from *A. brasilense* grown overnight in MMAB in the absence of ethylene or at the designated ethylene concentration. For the gene expression analysis of *glnZ, glnB, amtB*, and *nifH* cells were grown overnight in MMAB, washed and resuspended at an OD_600_ of 1.0 in MMAB without a nitrogen source and grown without shaking for 24 hours in the presence or absence of ethylene at the indicated concentration. RNA isolation was performed using the TRIzol:chloroform method with some modifications. Briefly, 2 mL of cells were pelleted before resuspension in 550 µL of TRIzol and dissolution of cell pellets. After cell pellets were dissolved, 250 µL of lysozyme was added, and samples were incubated for 20 min at 37°C. After incubation, the samples were placed on ice for 5 min before the addition of 200 µL chloroform. cDNA synthesis was performed using the Sensifast cDNA synthesis kit. RNA was normalized to 100 ng µL^-1^ and 100 ng µL^-1^ cDNA was prepared. qPCR was performed using a SYBR-NOROX kit. Primers for quantitative PCR were designed using Integrated DNA Technologies and gene expression levels relative to the previously validated reference genes *GlyA* and *RecA* [119] calculated following the 2^-∆∆ct^ method [120]. The data were then normalized to the expression in the absence of ethylene. The primers used to quantify *Azoetr1*, *Azorr*^*etr1*^, *OH82-RS30850*, and *OH82_RS14895* are listed in S5 Table. All other primers used were from prior studies [49,121,122].

### RNA-seq

To determine the downstream targets of the ethylene signaling pathway, we conducted an RNA-seq experiment in which cells were grown in liquid culture in MMAB with NaNO_3_ for 16 h and then for 4 h in the presence or absence of 100 ppb ethylene. RNA was extracted as previously described. RNA-seq and bioinformatics analyses were performed using GeneWiz (South Plainfield, NJ, USA). The Wald test was used to generate *p* values. Genes with adjusted *p* values ≤ 0.05 and absolute log_2_ fold changes ≥ 1 were considered differentially expressed. Gene Ontology categories were determined using the Kyoto Encyclopedia of Genes (KEGG). The RNA-seq data discussed in this publication have been deposited in the NCBI GEO Omnibus [123] with GEO Series Accession Number GSE179776 (https://www.ncbi.nlm.nih.gov/geo/query/acc.cgi?acc=GSE179776).

### Immunoblots

Proteins from membranes isolated from *A. brasilense* were separated by SDS-PAGE. Wild-type and mutant AzoEtr1 protein expression were analyzed with immunoblots using a 1:1000 dilution of a polyclonal anti-GFP antibody (Abcam). An infrared IRDye800 (LI-COR) secondary antibody was used, and detected using an Odyssey CLx Imager (LI-COR).

### Metabolomics

Samples were grown in TY overnight before being washed in Chemotaxis (Che) Buffer, and the OD_600_ was normalized to 1.0. These samples were used to inoculate 10mL of MMAB supplemented with NaNO_3_. The samples were grown for 24 h in sealed tubes injected with 100 ppb ethylene or an equivalent volume of ethylene-free air. After 24 h of growth, the cells were pelleted by centrifugation, the supernatant was removed, and the samples were flash-frozen in liquid nitrogen before analysis by LC-MS/MS. Partial least squares differential analysis was performed to determine the separation of the metabolic profiles. The analysis was performed at the Biological and Small Molecule Mass Spectrometry Core Facility in the Department of Chemistry at the University of Tennessee. MetaboAnalyst 5.0 was used for partial least squares discriminant analysis and to conduct pathway analysis [124]. Data represent the analysis of three biological replicates for each condition which were each analyzed with three technical replicates and normalized to tissue fresh weight. Pathway analysis of metabolites was performed using the Kyoto Encyclopedia of Genes (KEGG) (https://www.genome.jp/kegg/).

### PHB staining

Cells were grown overnight in TY medium and washed with Che Buffer before adjusting the OD_600_ to 1.0 in Che buffer [118]. These samples were used to inoculate 50 mL MMAB supplemented with 10 mM NaNO_3_. The cultures were grown in flow-through chambers and treated with either 100 ppb ethylene or ethylene-free air. Stirring was achieved using a stir bar set to 200 RPM. Growth was allowed to occur for 24 h after which 2 mL of these cultures were collected and washed with Che Buffer before staining with 0.5% (w/v) Nile Red for 30 min., and then imaged using a Zeiss Axio Observer Z1 microscope. Images were analyzed using ImageJ/Fiji (ver. 1.52E) to determine the number of foci per cell. The statistical significance of the differences in the median values was determined using the Mann-Whitney Test.

### Oxidative stress assays

Sp7 and AzoEtr1::Tet^R^ cells were grown on 1.5% (w/v) TY agar for 24 h at which time a 6 mm filter paper disk soaked in 0.3% (v/v) hydrogen peroxide was placed in the center of the agar dish. After 24 h, the zone of inhibition diameter was measured.

### Carotenoid extraction

Cultures were grown for three days in TY medium at 28°C at which time cells were pelleted and resuspended in an equal volume of 100% (v/v) methanol. The samples were shaken overnight at room temperature at a speed of 180 RPM. The OD_485_ of the supernatant was determined using an Evolution 60 spectrophotometer (Thermo Scientific).

### Colony morphology

The cells were grown overnight in TY medium and adjusted to an OD_600_ of 1.0. 10µL spots were made on TY 1.5% (w/v) agar plates and allowed to dry and grow for three days before imaging with an Olympus SZH10 microscope using a Canon EO5 camera.

### Aerotaxis Assay

Aerotaxis assays were performed as previously described with some changes [67]. Cells were grown to an OD_600_ of 0.6 in MMAB and washed in Che buffer before resuspension to an OD_600_ of 0.6. An optically flat capillary tube was then used to draw cells from the cell suspension. This capillary tube was placed in focus under a microscope and a flow nitrogen gas was used for 3 minutes to abolish existing oxygen gradients. The gas flow was then switched to air and band formation was visualized under a light microscope at 4x.

### Statistics

ANOVA and Mann-Whitney tests were performed using GraphPad Prism (ver. 10.0.2). Student’s t-test was performed done using Microsoft Excel.

## Supporting information

S1 Fig Predicted amino acid sequences of AzoEtr1 and Azorr^etr1^. (PDF)

S2 Fig Genomic structure around *Azoetr1* in *A. brasilense* Sp7. (PDF)

S3 Fig Nucleotide sequence of plasmid (ABSP7_p1) containing *Azoetr1* and *Azorr* ^*etr1*^. (PDF)

S4 Fig Alignment of AzoEtr1 homologs from different *Azospirillum* species. (PDF)

S5 Fig Ethylene and the AzoEtr1::TetR disruption reduces biofilm formation. (PDF)

S6 Fig Additional traits affected by disruption of AzoEtr1. (PDF)

S7 Fig Transcript abundance of *Azorr* ^*etr1*^ in AzoEtr1::Gm^R^ lines. (PDF)

S8 Fig Empty vector plasmids do not rescue biofilm formation and responses to ethylene. (PDF)

S9 Fig Ethylene measurements on *A. brasilense.* (PDF)

S10 Fig Root colonization of tomato is inhibited by ethylene or disruption of AzoEtr1. )
(PDF)

S11 Fig Principal Component Analysis (PCA) of global gene transcript changes caused by ethylene. (PDF)

S12 Fig Transcript abundance of *Azoetr1* and *Azorr* ^*etr1*^ in response to ethylene. (PDF)

S1 Table Genes predicted to be co-transcribed in response to ethylene that are associated with one or more of the top 36 gene transcripts altered by ethylene. (PDF)

S2 Table Genes predicted to be co-transcribed in response to ethylene but, not included in the top 36 genes altered by ethylene. (PDF)

S3 Table Metabolites significantly altered by treatment with ethylene. (PDF)

S4 Table Cloning primers. (PDF)

S5 Table qPCR primers. (PDF)

S1 Data Differentially expressed genes. (XLSX)

S2 Data Underlying numerical data. (XLSX)
