# Supplementary material for: Ethylene signals through an ethylene receptor to modulate biofilm formation and root colonization in a beneficial plant-associated bacterium
Source: PLoS Genet. 2025 Feb 7;21(2):e1011587. doi: 10.1371/journal.pgen.1011587 (PMC11819568; doi:10.1371/journal.pgen.1011587)
Supplement: S1 Fig — (PDF) [file pgen.1011587.s001.pdf]

Supplemental Figures

A

```
MFGGVEAFFDTSAYLPHGVCLFWRPEILTLHIVSDVLTGLSYYSIPVALLYFVVKRRDVAFTWIVWLFAA
FILACGTTHFFSLWTLWYPDYAVEGIVKALTAMVSVLTAVALWVQMPKALALPSATQLADANGALQREIE
IRRQAELRYASFFNNLAEGLFVVTVLPDGDFAFDTLNPAHARGTGIDPETIRGRLVREAVPPETAAAVIE
RYSACVAAGGPIDYEETLDLPVGRRTWHTVLVPVRGEGGEVVQILGSSRDITDRKRLQEELVQTSKLATL
GTLAAGMAHEMSQPLNIIRIWAENALSRLRDGDTDTARLDKVLTIMSDQAERMGRIIDHMRTFSRRDGAT
QRFDPAASVRSAVELVSNQFALENIEVVSDVPAIDCVTRGRPLQLEQVLVNLLSNARDAILEWRADPDGS
PAAGRIAIGMRCDMTAGRAVITITDDGGGIDPDILPRIFDPFFTTKEVGKGSGLGLSIGYGIIDSMGGRI
DAANVTQDDGSRGVRFTITVPVSHPSIQDVERAHA
```

B

```
MPDPVTAPLHVLVAEDEALAAMALEDFLSRKGYRVTLAQDGQEGLERYSADPADLVITDLRMPRMDGRAL
IRELRIKAAGLPILVMTGFLSMETGEDDLTSDRWQPLVVLRKPVSPQVILDTLANLAQAA
KLRPA
```

S1 Fig. Predicted amino acid sequences of AzoEtr1 and Azorr^{etr1}. A) AzoEtr1 is predicted to be 525 amino acids long and **B)** Azorr^{etr1} to be 135 amino acids long.
