# Supplementary material for: Ethylene signals through an ethylene receptor to modulate biofilm formation and root colonization in a beneficial plant-associated bacterium
Source: PLoS Genet. 2025 Feb 7;21(2):e1011587. doi: 10.1371/journal.pgen.1011587 (PMC11819568; doi:10.1371/journal.pgen.1011587)
Supplement: S2 Fig — (PDF) [file pgen.1011587.s002.pdf]

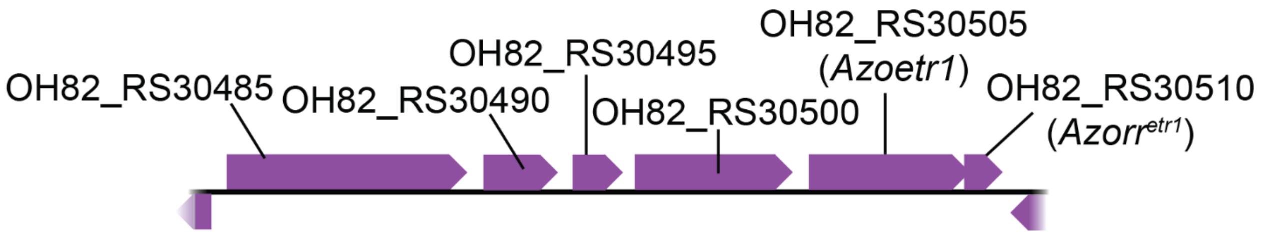

S2 Fig. Genomic structure around *Azoetr1* in *A. brasilense* Sp7. Downstream is OH82_RS30510 we call *Azorr*^{etr1} which is predicted to encode a response regulator protein. Upstream, OH82_RS30500 is predicted to encode a peptide chain release factor 3, OH82_RS30495 a hypothetical protein, OH82_RS30490 an aspartate-semialdehyde dehydrogenase, and OH82_RS30485 a maltodextrin or glycogen phosphorylase.
