# Supplementary material for: Ethylene signals through an ethylene receptor to modulate biofilm formation and root colonization in a beneficial plant-associated bacterium
Source: PLoS Genet. 2025 Feb 7;21(2):e1011587. doi: 10.1371/journal.pgen.1011587 (PMC11819568; doi:10.1371/journal.pgen.1011587)
Supplement: S3 Fig — (PDF) [file pgen.1011587.s003.pdf]

GCTATCTATACGTCCGAACGAAGAGGGCCGTGGGGCGTTCCGCCTCCCGGCCCTCTTCGCCGTTGTCTGCACCGCTG
AGTTACGGGAACGCGCATGTCCGAGCTTCTGGACGCTGTTTCCCGGCGTCGAACCTTCGCGATCATCGCGCACCCCG
ACGCCGGCAAGACCACCCTCACCGAAAAGCTGCTGCTGTTCGGCGGCGCCATCCAGATGGCCGGCGCCGTGAAGGCC
CGCGGCGAGCAGCGCCGCGCCAAGTCGGACTGGATGAAGGTGGAGCGCGAGCGCGGCATCTCCGTGACCGCCTCCGT
CATGACCTTCGATTACGAAGGGCGCACCTTCAACCTGCTGGACACGCCGGGCCACGAGGACTTCTCGGAGGACACCT
ACCGGACGCTGACCGCGGTGGACAGCGCCGTCATGGTGATCGACGGCGCGAAGGGCATCGAAAGCCAGACCTTGAAG
CTGTTCGAGGTCTGCCGCCTGCGCGACGTGCCGATCATCACCTTCTGCAACAAGATGGACCGCGAGGCCCGCGACCC
CTTCGACCTCATCTCCGAGATCGAAAGCTCGCTGGCGCTGGAGGTGACCCCGGCGAGCTGGCCCATCGGCATGGGCC
GCGACTTCCTGGGCTGCTACGATCTGATCCGCGACCGCCTGATCCTGATGGATCGCACCAAGGGCGACGAGATCGAC
GACGGCATCGAGTGCAACGGTCTCGACGACCCGCGCCTCGACGATCTGCTTCCAGCCCACGCGGTGGCGAAGCTGCG
CGAGGAGGTGGAGATGGCCCGCGGGCTGATGCCGCCCTTCGACCTGGAGGCCTACCGCGCCGGCCATCTGACGCCGA
TTTACTTCGGCTCGGCCATCAACAACTTCGGCGTGCGCGAGCTTCTGCAGGGCCTCGCTGAGAACGCCCCGCCGCCG
CGCCCCCAGCCGGCGGAGCAGCGCTCCGTGCAGCCCGACGAGGGCAAGTTCAGCGGCTTCGTGTTCAAGGTCCAGGC
CAACATGGACCCCAACCACCGCGACCGCATCGCCTTCGTGCGCATCTGCTCGGGCAAGTTCAAGCGCGGTGCCAAGC
TGAAGCATGTCCGTTCGGGCAAGCTGATGGCGGTGAACAACGCCGTGCTGTTCCTGGCCCGCGACCGCGAGCTGGCG
GAAGAGGCGTGGCCCGGCGACATCATGGGCATCCCGAACCACGGGTCCTTGCGCATCGGCGACACGCTGACCGAGGG
CGAGGACCTGCGCTTCACCGGCGTGCCGAGCTTCGCACCGGAACTGCTGCAGCGCGTCCGGCTGGAAGACCCGATGC
GCGTGAAGCACCTGCGCAAGGCGCTGGAGCATTTCGCGGAGGAAGGCGCCTCCCAGGTGTTCAAGCCGCTGACCGGG
GCGGATTGGGTGGTCGGCGTGGTCGGCCAGCTGCAGTTCGAGGTGCTGGCCGCCCGCATCGAGGCGGAATACGGTCT
CGCCGCCCGCTTCGAGGGAGCGGGTGTGGACGCCGCCCGCTGGATCGAGACGGACGACGAGGACCAACTCAAGAAGT
TCCTCGACCTCAATCGCTCGGCAGCGGCGGAGGACCATGATGGGGCGCTGGTGTTCCTCGCCCGCAACGCTTGGCAC
CTGAACCGCACGCAGGAGGATTTTCCGGCCCTGCGCTTCTTGAAGACCCGCGAGCAAAACCGCAAGGTGCACACTGC
GGCGTAACGTGCATCCAGAACCATGCGGAACGCGCTGACCATTGGCCGGCATCGCGTTCCGCGTGTTGCTCCGCCCC
GCTGATTCGAGTAGCGTTTGCTGACGCAGCCTCCGCGGCATGGTGCCGCAAGGCGTTGTGCTTCAAGGTTTTGGGGC
AAGATCAGAGAAACGGTGGCATTTCCGGACCCGGCTGGACCGCGGACGGTGACCGAATAAAAGAGGGGCGGCATGTT
CGGTGGCGTGGAAGCCTTCTTCGACACCAGCGCGTACTTGCCTCACGGCGTGTGCCTGTTCTGGCGTCCGGAAATCC
TGACCCTGCACATCGTGTCCGATGTGTTAACCGGCCTCTCCTATTATTCGATTCCGGTGGCGCTGCTTTATTTCGTT
GTGAAGCGCCGCGACGTGGCCTTCACTTGGATCGTCTGGCTGTTCGCCGCCTTCATCCTGGCCTGCGGCACGACGCA
TTTCTTCAGCCTGTGGACCTTGTGGTATCCCGACTATGCCGTGGAAGGCATCGTCAAGGCGCTGACCGCAATGGTGT
CGGTGCTGACCGCCGTCGCCTTGTGGGTGCAGATGCCCAAGGCTCTCGCTTTGCCCAGCGCCACCCAGCTCGCCGAC
GCCAACGGAGCCCTTCAGCGCGAGATCGAGATCCGGCGTCAGGCCGAGCTGCGCTACGCCAGCTTCTTCAACAATCT
GGCGGAGGGGCTGTTCGTCGTCACGGTGCTGCCGGACGGTGACTTCGCCTTCGACACGCTGAACCCCGCCCACGCGC
GGGGGACGGGAATCGACCCGGAGACCATCCGCGGCCGGCTGGTCCGCGAGGCCGTTCCGCCGGAGACTGCCGCGGCG
GTGATCGAACGCTACAGCGCCTGCGTCGCTGCGGGCGGCCCGATCGATTACGAGGAGACGCTGGACCTTCCGGTCGG
ACGGCGCACGTGGCACACCGTCCTGGTGCCGGTGCGCGGCGAGGGAGGGGAGGTGGTCCAGATTCTGGGCAGCTCCC
GCGACATCACCGACCGCAAGAGGCTTCAGGAGGAGCTGGTCCAGACCTCCAAGCTGGCGACCCTGGGCACGCTTGCC
GCCGGCATGGCCCATGAGATGAGCCAACCCCTGAACATCATCCGGATCTGGGCCGAGAACGCCCTGTCCCGCCTGCG
CGACGGCGACACCGACACCGCGCGCCTGGACAAGGTGCTGACCATCATGTCGGACCAGGCGGAGCGCATGGGCCGCA
TCATCGACCATATGCGCACCTTCAGCCGCCGCGACGGCGCCACCCAGCGCTTCGACCCCGCGGCCAGCGTCCGCTCC
GCCGTCGAGTTGGTGTCCAACCAGTTCGCCTTGGAAAACATCGAGGTCGTGAGCGACGTCCCGGCCATCGACTGCGT
CACGCGCGGCCGTCCCCTGCAACTGGAGCAGGTGCTGGTCAATCTGCTGTCCAACGCGCGCGATGCGATTCTGGAAT
GGCGCGCCGACCCCGACGGGTCGCCCGCTGCCGGCCGGATTGCCATCGGGATGCGTTGCGACATGACCGCCGGGCGG
GCCGTCATCACCATCACCGACGACGGCGGCGGCATCGACCCGGACATCCTGCCGCGCATCTTTGACCCCTTCTTCAC
GACCAAGGAAGTGGGGAAGGGGTCGGGCCTCGGACTGTCGATCGGCTATGGCATCATCGACTCCATGGGGGGCCGGA
TCGATGCGGCGAATGTGACCCAAGACGATGGAAGCCGCGGCGTCCGTTTCACCATCACCGTTCCGGTGTCCCATCCC
TCCATTCAGGACGTGGAGCGCGCTCATGCCTGACCCCGTCACCGCTCCGTTGCATGTTCTGGTGGCCGAGGACGAAG
CCTTGGCCGCCATGGCGTTGGAGGATTTTCTTTCCCGCAAGGGATACCGCGTGACCCTGGCCCAGGACGGGCAGGAG
GGGTTGGAGCGCTACAGCGCCGACCCCGCCGACCTCGTCATCACCGATCTGCGCATGCCGCGTATGGACGGCCGAGC
CCTGATCCGCGAGCTGAGGATCAAGGCCGCCGGACTGCCGATCCTGGTGATGACCGGCTTCCTGTCGATGGAGACGG
GGGAGGACGACCTGACGTCCGACCGATGGCAACCACTGGTGGTGTTGCGCAAGCCGGTCAGTCCTCAGGTCATCCTG
GACACGCTCGCCAACCTCGCCCAGGCGGCGAAGCTGCGGCCTGCATGATCGCGCCTTCCTAGAACCGGTCGCGCAGT
TCCGGCAGGGGGACGATGCGGACGCCCTTGGCCTCCAGGGTGCGGCGCAGGTTTCCGGCCATGGCAACGGCGCTCGG
TTCGTCGCCATGGACGCAGACGGAATGGACGGTGCAGGGGATGCGCGTGCCGGTGACCGACAGGATCACGCCCTCCT
CCAGCATGCGCAGGACGTTGGCGGCCGCCACGTCCGGGTCATGCACCATCGCGCCGGGCTGACCGCGCGGCACGAGG
TTGCCGTTGTCGTCGTAGGCGCGGTCGGCAAAGACCTCCTCCGCCGTGGGCAGGCCCAGCGTCCGCCCGGCCCGAGC
CATTTCCGACCCGGCGGTGGCG

S3 Fig. Nucleotide sequence of plasmid (ABSP7_p1) containing *Azoetr1* and *Azorr*^{etr1}.. Green highlights the predicted RpoN binding site in the *Azoetr1* promoter, Cyan highlights *Azoetr1*, yellow *Azorr*^{etr1}, and red the overlap between the stop codon of *Azoetr1* and the start codon of *Azorr*^{etr1}.
