# Supplementary material for: Ethylene signals through an ethylene receptor to modulate biofilm formation and root colonization in a beneficial plant-associated bacterium
Source: PLoS Genet. 2025 Feb 7;21(2):e1011587. doi: 10.1371/journal.pgen.1011587 (PMC11819568; doi:10.1371/journal.pgen.1011587)
Supplement: S4 Fig — (PDF) [file pgen.1011587.s004.pdf]

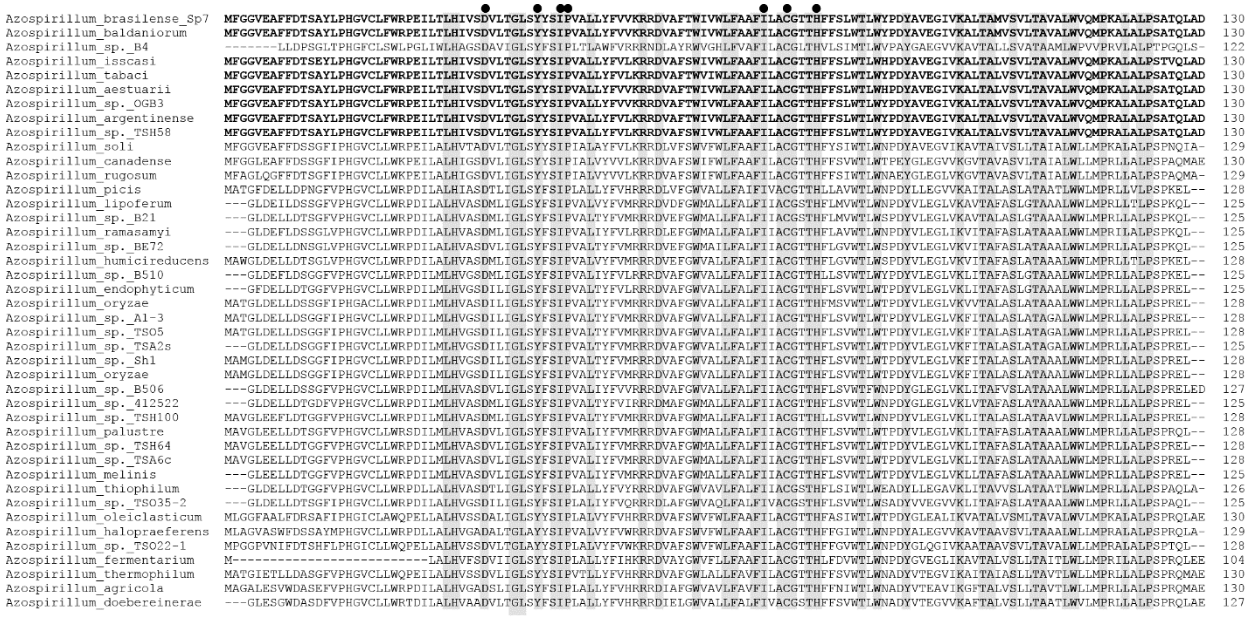

S4 Fig. Alignment of AzoEtr1 homologs from different *Azospirillum* species. The first 130 amino acids of AzoEtr1 in *A. brasilense* Sp7 compared to homologs from other *Azosprillum* species. Based on BLAST search conducted 6 July 2024 against the *Azosprillum* genus. Black dots over sequences mark amino acids known to be critical for ethylene binding to AtETR1 from *A. thaliana*. Grey highlights identical residues. All sequences contain these seven residues. Sequences in bold are 100% identical in this region of the protein to AzoEtr1 from *A. brasilense* Sp7.
