# Supplementary material for: Ethylene signals through an ethylene receptor to modulate biofilm formation and root colonization in a beneficial plant-associated bacterium
Source: PLoS Genet. 2025 Feb 7;21(2):e1011587. doi: 10.1371/journal.pgen.1011587 (PMC11819568; doi:10.1371/journal.pgen.1011587)
Supplement: S5 Fig — (PDF) [file pgen.1011587.s005.pdf]

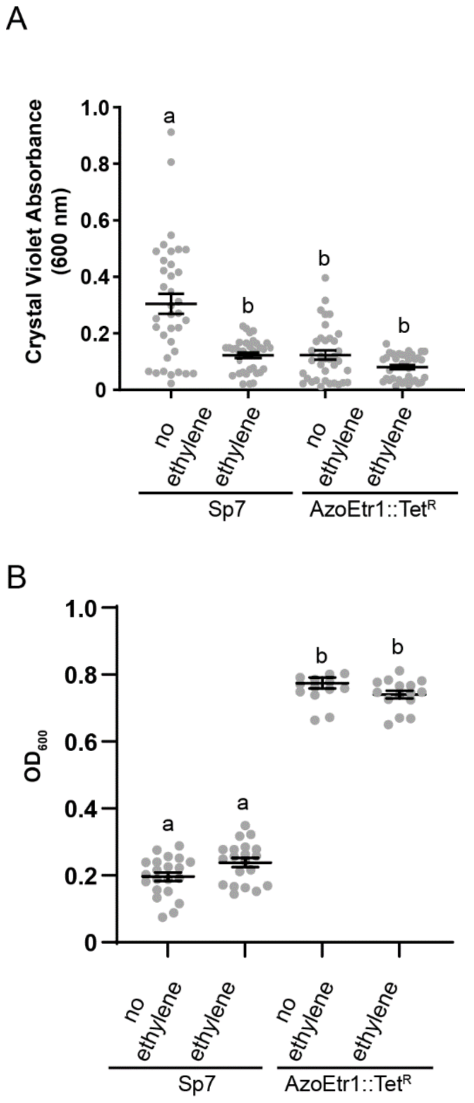

S5 Fig. Ethylene and the AzoEtr1::TetR disruption reduces biofilm formation. Cells were kept in ethylene-free air or 100 ppb ethylene for 3 days. **A)** Biofilm formation of wild-type (Sp7) and the AzoEtr1::Tet^R disruptant line were assayed with crystal violet staining. **B)** Total cell growth was evaluated by measuring the OD_{600} of the planktonic culture in each assay well in samples treated as in A. Data is the average ± SEM. Different letters denote statistically different *p* value ≤ 0.05 as determined by ANOVA and shows that addition of ethylene and disruption of AzoEtr1 have statistically indistinguishable effects on biofilm formation under these conditions but disruption of AzoEtr1 leads to increased total cell growth. Growth data of wild-type is from figure 2B.
