# Supplementary material for: Ethylene signals through an ethylene receptor to modulate biofilm formation and root colonization in a beneficial plant-associated bacterium
Source: PLoS Genet. 2025 Feb 7;21(2):e1011587. doi: 10.1371/journal.pgen.1011587 (PMC11819568; doi:10.1371/journal.pgen.1011587)
Supplement: S6 Fig — (PDF) [file pgen.1011587.s006.pdf]

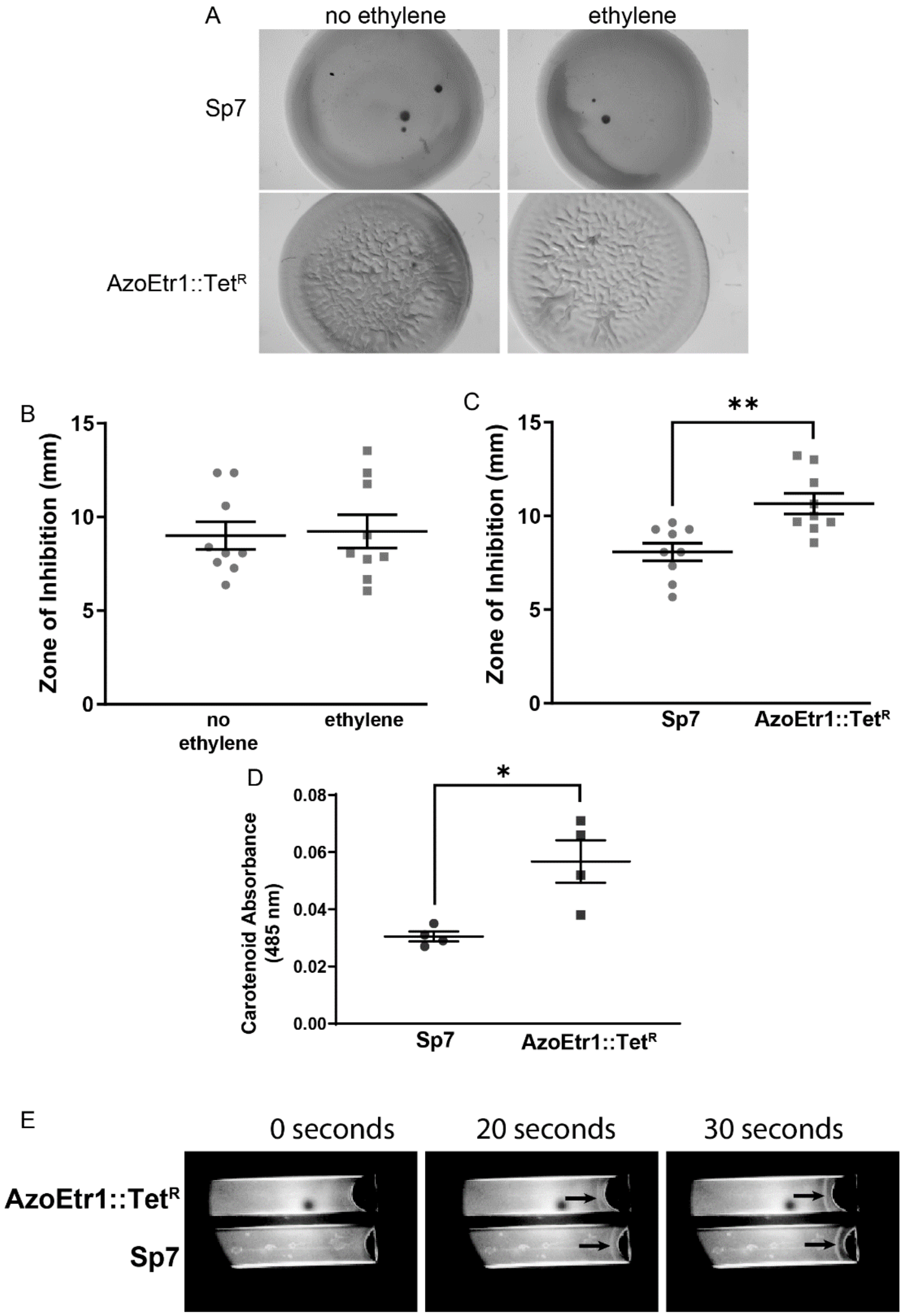

S6 Fig. Additional traits affected by disruption of AzoEtr1. Disruption of AzoEtr1 resulted in **A)** corrugated colony morphology and **B)** increased susceptibility to H_2O_2 stress, whereas, treatment with 100 ppb ethylene did not affect these traits (A,C). Disruption of *Azoetr1* **D)** increased carotenoid levels but did not affect **E)** aerotaxis. Arrow indicates location of band of motile bacteria. B-D, Data is the average ± SEM; * *p* value < 0.05 and ** *p* value < 0.01 compared to control as determined by Student's t-test.
