# Supplementary material for: Ethylene signals through an ethylene receptor to modulate biofilm formation and root colonization in a beneficial plant-associated bacterium
Source: PLoS Genet. 2025 Feb 7;21(2):e1011587. doi: 10.1371/journal.pgen.1011587 (PMC11819568; doi:10.1371/journal.pgen.1011587)
Supplement: S7 Fig — (PDF) [file pgen.1011587.s007.pdf]

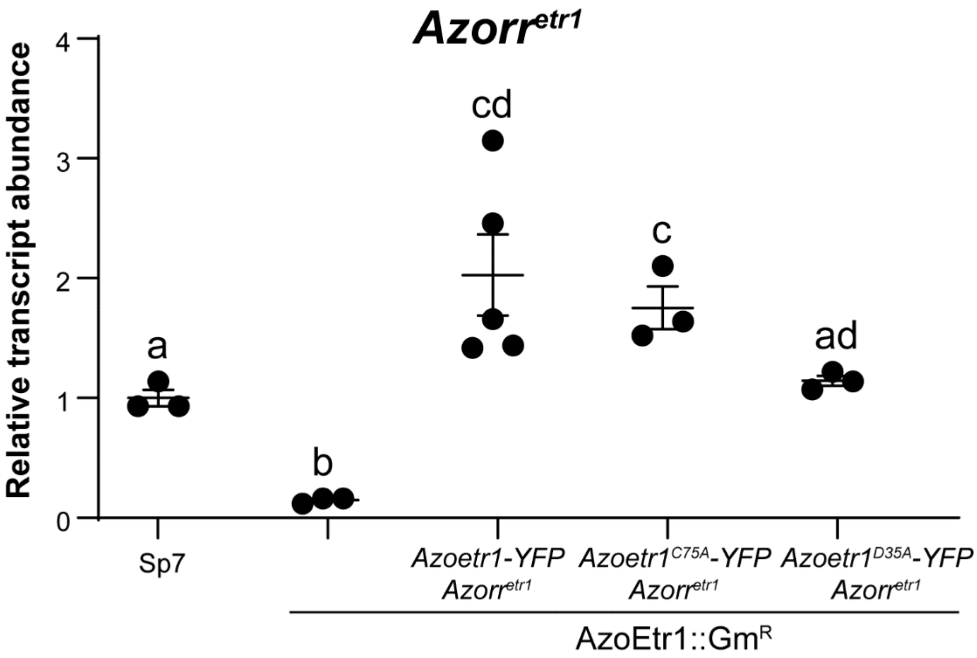

S7 Fig. Transcript abundance of *Azorr*^{etr1} in AzoEtr1::Gm^{R} lines.

AzoEtr1::Gm^{R} was transformed with the indicated construct expressing either wild-type or mutant *AzoEtr1-YFP* as described in the materials and methods. These lines were also transformed with *Azorr*^{etr1} on a second plasmid and the transcript abundance of *Azorr*^{etr1} was determined. Wild-type (Sp7) and AzoEtr1::Gm^{R} are included for comparison. Data were normalized to housekeeping genes as noted in the materials and methods and then to Sp7. Data is the average ± SEM. Different letters denote statistical difference (p value < 0.05) using ANOVA.
