# Supplementary material for: Ethylene signals through an ethylene receptor to modulate biofilm formation and root colonization in a beneficial plant-associated bacterium
Source: PLoS Genet. 2025 Feb 7;21(2):e1011587. doi: 10.1371/journal.pgen.1011587 (PMC11819568; doi:10.1371/journal.pgen.1011587)
Supplement: S8 Fig — (PDF) [file pgen.1011587.s008.pdf]

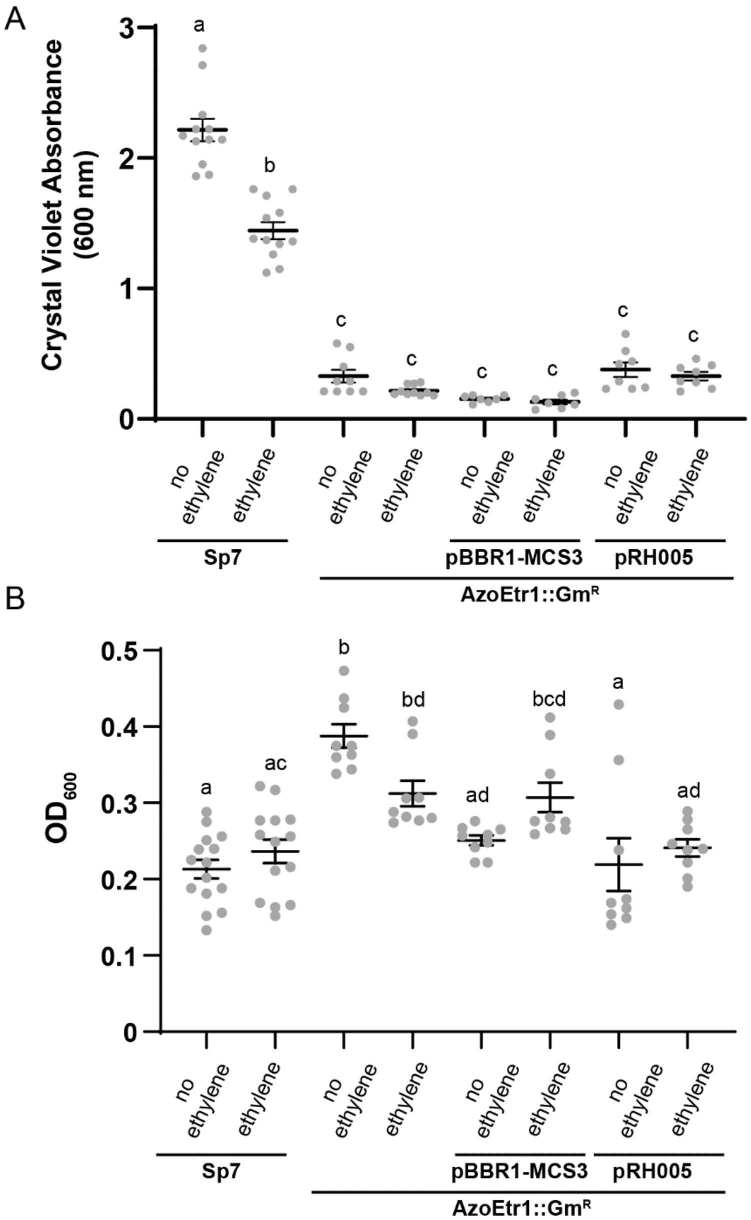

S7 Fig. Empty vector plasmids do not rescue biofilm formation and responses to ethylene. **A)** AzoEtr1::Gm^R was transformed with either pBBR1-MCS3 or pRH005 vector and biofilm assessed as described in the materials and methods. Data for wild-type (Sp7) shown as a control. **B)** Total cell growth was evaluated by measuring the OD_{600} of the planktonic culture in each assay well in samples treated as in A. Data is the average ± SEM. Different letters denote statistically different p value ≤ 0.05 as determined by ANOVA. Growth data for wild-type and AzoEtr1::Gm^R are from figure 2.
