# Supplementary material for: Ethylene signals through an ethylene receptor to modulate biofilm formation and root colonization in a beneficial plant-associated bacterium
Source: PLoS Genet. 2025 Feb 7;21(2):e1011587. doi: 10.1371/journal.pgen.1011587 (PMC11819568; doi:10.1371/journal.pgen.1011587)
Supplement: S9 Fig — (PDF) [file pgen.1011587.s009.pdf]

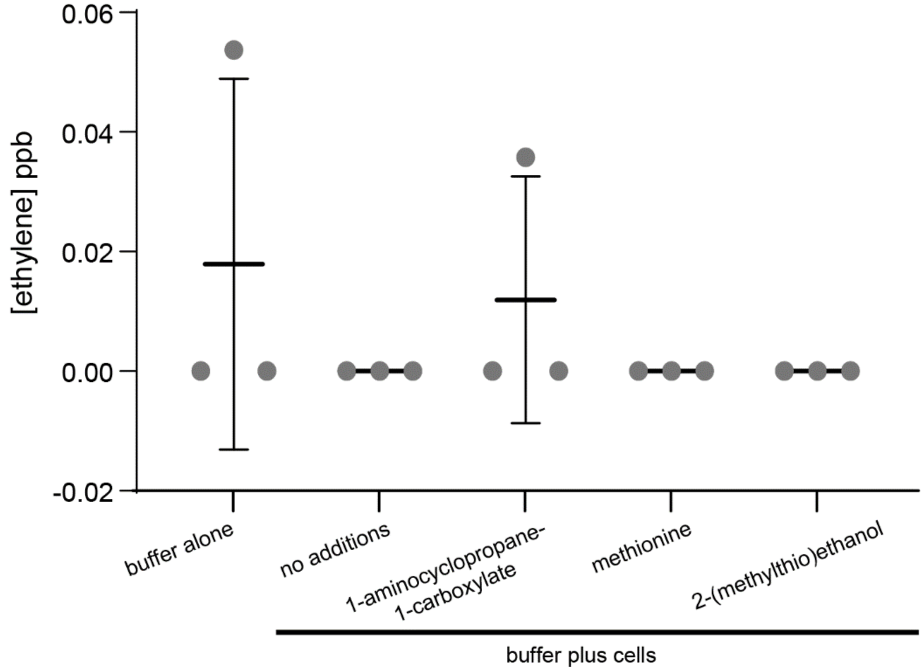

S8 Fig. Ethylene measurements on *A. brasilense*. Cultures of *A. brasilense* were grown overnight in sealed tubes in the presence or absence of chemicals known to enhance ethylene production by some bacterial species (see text). At that time, the concentration of ethylene was determined in the headspace using an ETD-300 ethylene analyzer. None of the samples tested gave ethylene production significantly above buffer alone. Data is the mean ± SD.
