# Supplementary material for: Ethylene signals through an ethylene receptor to modulate biofilm formation and root colonization in a beneficial plant-associated bacterium
Source: PLoS Genet. 2025 Feb 7;21(2):e1011587. doi: 10.1371/journal.pgen.1011587 (PMC11819568; doi:10.1371/journal.pgen.1011587)
Supplement: S10 Fig — ) (PDF) [file pgen.1011587.s010.pdf]

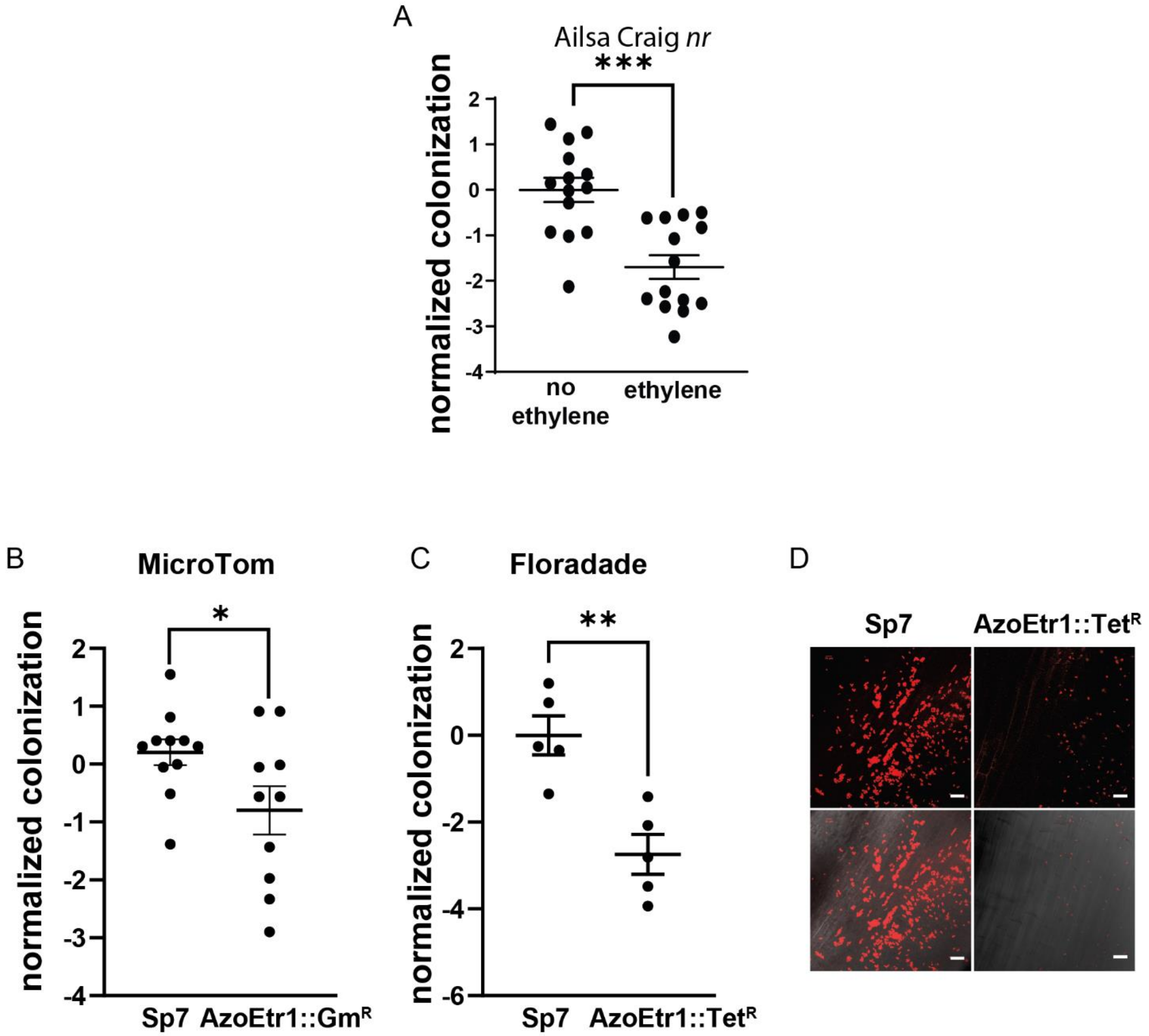

S9 Fig. Root colonization of tomato is inhibited by ethylene or disruption of AzoEtr1. Colonization assays of tomato roots were carried out as described in the materials and methods for 24 h. **A)** Assays were carried out on the ethylene insensitive *never ripe* (*nr*) mutant in the Ailsa Craig background in the presence or absence of 100 ppb ethylene. Colonization of **B)** MicroTom and **C)** Floradade was evaluated with wild-type (Sp7) or insertional mutants of AzoEtr1 as designated. A-C, Biofilm was measured with crystal violet assays and normalized as described in the materials and methods. Data is the average ± SEM; * *p* value < 0.05; ** *p* value < 0.01, *** *p* value < 0.001 as compared to the no ethylene controls determined by Student's t-test. **D)** Representative images of colonization of Floradade roots by YPF-expressing *A. brasilense*. Wild-type (Sp7) compared to the AzoEtr1::Tet^R insertional mutant. Top images - fluorescent image, bottom merged with DIC. Scale bars = 20 µM
