# Supplementary material for: Ethylene signals through an ethylene receptor to modulate biofilm formation and root colonization in a beneficial plant-associated bacterium
Source: PLoS Genet. 2025 Feb 7;21(2):e1011587. doi: 10.1371/journal.pgen.1011587 (PMC11819568; doi:10.1371/journal.pgen.1011587)
Supplement: S11 Fig — (PDF) [file pgen.1011587.s011.pdf]

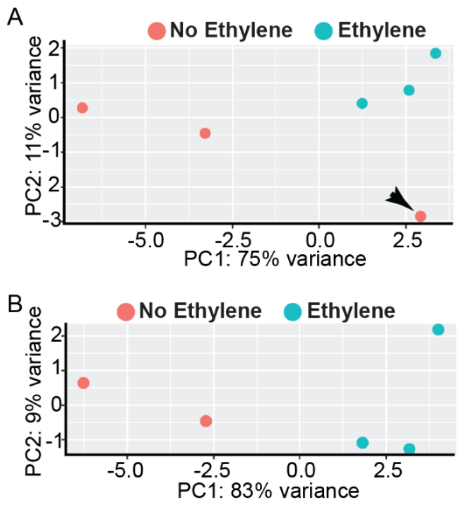

S10 Fig. Principal Component Analysis (PCA) of global gene transcript changes caused by ethylene. *A. brasilense* cells were grown in liquid culture and samples prepared as described in the main text. **(A)** PCA was used to compare the variance of the samples and showed that one control sample (marked with an arrow) separated from the other 2 controls. Because of this we removed this sample from further analysis and generated a **(B)** separate PCA plot.
