# Supplementary material for: Ethylene signals through an ethylene receptor to modulate biofilm formation and root colonization in a beneficial plant-associated bacterium
Source: PLoS Genet. 2025 Feb 7;21(2):e1011587. doi: 10.1371/journal.pgen.1011587 (PMC11819568; doi:10.1371/journal.pgen.1011587)
Supplement: S12 Fig — (PDF) [file pgen.1011587.s012.pdf]

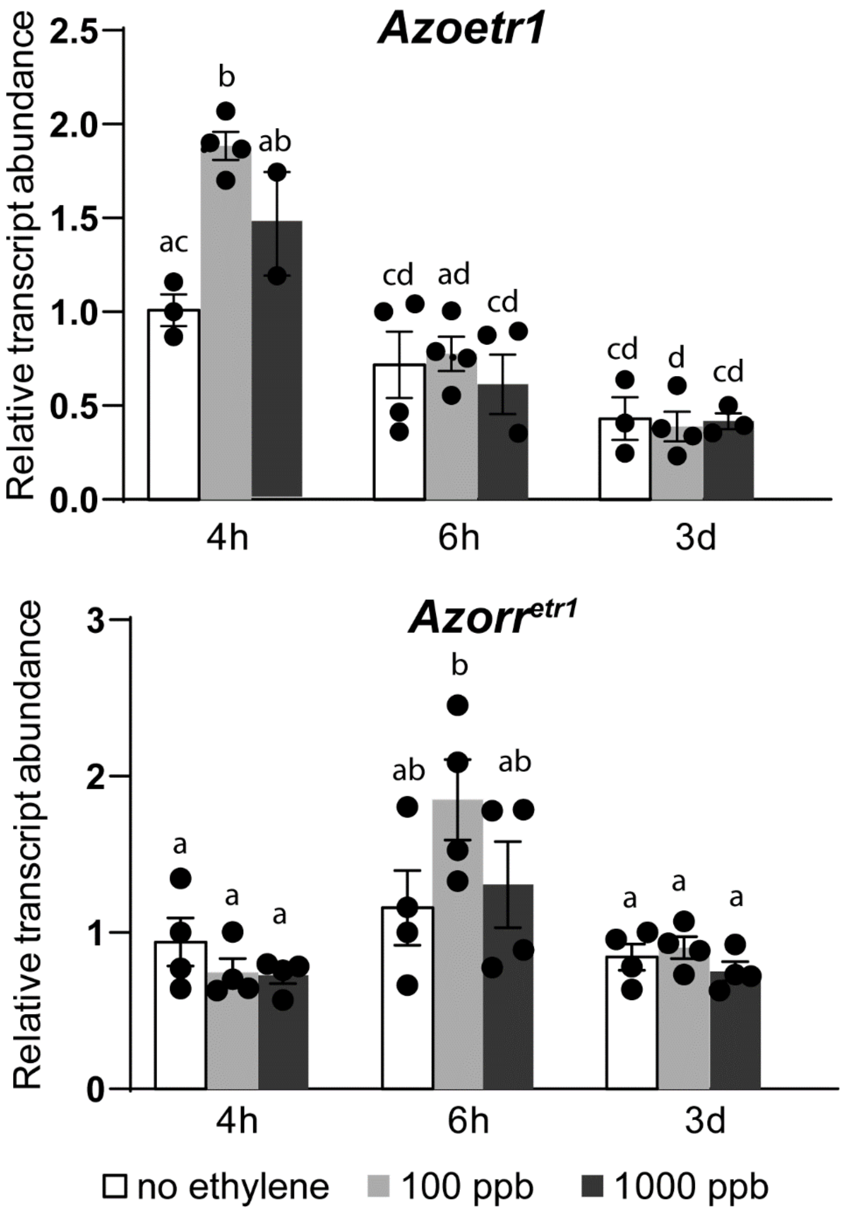

S12 Fig. Transcript abundance of *Azoetr1* and *Azorr*^{etr1} in response to ethylene. *A. brasilense* cells were treated with the indicated concentration of ethylene for the indicated period of time. Data were normalized to housekeeping genes as noted in the materials and methods and abundance of the gene in the absence of added ethylene at the indicated time and represents 4 biological replicates with three technical replicates. Data is the average ± SEM. Different letters denote statistical difference (p value < 0.05) at each time point using ANOVA.
