# Supplementary material for: Ethylene signals through an ethylene receptor to modulate biofilm formation and root colonization in a beneficial plant-associated bacterium
Source: PLoS Genet. 2025 Feb 7;21(2):e1011587. doi: 10.1371/journal.pgen.1011587 (PMC11819568; doi:10.1371/journal.pgen.1011587)
Supplement: S1 Table — (PDF) [file pgen.1011587.s013.pdf]

S1 Table. Genes predicted to be co-transcribed in response to ethylene that are associated with one or more of the top 36 gene transcripts altered by ethylene.[a]

locus tag	Log_2fold change	adjusted *p*-value	gene name	annotation
OH82_RS16030	1.17	1.09×10^{-9}	*ftsZ*	cell division protein FtsZ
OH82_RS16035	0.40	5.40×10^{-3}	*ftsA*	cell division protein FtsA
OH82_RS30845	1.38	4.69×10^{-15}		pyruvate dehydrogenase complex dihydrolipoamide acetyltransferase
OH82_RS30850	1.18	1.13×10^{-15}		pyruvate dehydrogenase complex E1 component subunit β
OH82_RS30855	0.47	1.01×10^{-4}		pyruvate dehydrogenase (acetyl-transferring) E1 component subunit α
OH82_RS07180	0.62	3.40×10^{-3}		hypothetical protein
OH82_RS07185	1.47	9.42×10^{-6}		dimethyl sulfoxide reductase anchor subunit
OH82_RS07190	0.79	1.60×10^{-2}		4Fe-4S dicluster domain-containing protein
OH82_RS07195	0.61	3.06×10^{-3}		molybdopterin-dependent oxidoreductase
OH82_RS07200	1.30	2.50×10^{-13}		CoA-binding protein
OH82_RS07205	1.33	2.37×10^{-16}		acetate--CoA ligase family protein
OH82_RS07210	1.71	5.56×10^{-6}		aldehyde dehydrogenase family protein
OH82_RS07215	1.37	1.77×10^{-3}		tripartite tricarboxylate transporter permease
OH82_RS07220	0.91	7.92×10^{-6}		tripartite tricarboxylate transporter TctB family protein
OH82_RS07225	0.53	1.39×10^{-3}		tripartite tricarboxylate transporter substrate binding protein
OH82_RS07230	0.99	2.66×10^{-6}		bifunctional enoyl-CoA hydratase/phosphate acetyltransferase
OH82_RS15310	0.66	3.07×10^{-15}		amidase
OH82_RS15315	0.74	1.76×10^{-5}		SDR family oxidoreductase
OH82_RS15320	0.46	1.56×10^{-3}		ABC transporter substrate-binding protein
OH82_RS15325	0.78	1.59×10^{-6}		branched-chain amino acid ABC transporter permease
OH82_RS15330	1.03	1.05×10^{-8}		branched-chain amino acid ABC transporter permease
OH82_RS15335	0.62	3.01×10^{-5}		ABC transporter ATP-binding protein
OH82_RS23770	-0.63	8.82×10^{-4}		hypothetical protein
OH82_RS23775	-1.20	5.74×10^{-16}		SCP2 sterol-binding domain-containing protein
OH82_RS29785	0.40	1.70×10^{-2}		LacI family DNA-binding transcriptional regulator
OH82_RS29790	1.09	4.54×10^{-8}		2-hydroxyacid dehydrogenase
OH82_RS29795	0.71	3.10×10^{-5}		amino acid ABC transporter ATP-binding protein
OH82_RS32100	0.50	1.75×10^{-3}		extracellular solute-binding protein
OH82_RS32105	0.48	1.23×10^{-2}		TerC family protein
OH82_RS32110	0.74	8.43×10^{-5}		tripartite tricarboxylate transporter permease
OH82_RS32115	1.06	1.02×10^{-7}		tripartite tricarboxylate transporter TctB family protein
OH82_RS32120	0.43	4.90×10^{-3}		hypothetical protein
OH82_RS32125	0.51	1.26×10^{-3}		tripartite tricarboxylate transporter substrate binding protein
OH82_RS06775	0.87	4.64×10^{-7}		hypothetical protein
OH82_RS06780	1.66	3.97×10^{-4}		hypothetical protein
OH82_RS06785	0.85	8.31×10^{-5}		flagellar hook-length control protein FliK
OH82_RS07360	0.64	9.21×10^{-4}		PTS transporter subunit EIIC
OH82_RS07365	1.46	1.39×10^{-4}	*pfkB*	1-phosphofructokinase
OH82_RS07370	0.58	3.72×10^{-2}	*ptsP*	phosphoenolpyruvate-protein phosphotransferase
OH82_RS19240	1.05	4.67×10^{-4}		ABC transporter permease subunit

OH82_RS19245	0.91	1.05×10^{-5}		ABC transporter ATP-binding protein
OH82_RS19250	0.64	8.00×10^{-4}		polyamine ABC transporter substrate-binding protein
OH82_RS19255	0.72	4.31×10^{-5}		enoyl-CoA hydratase/isomerase family protein
OH82_RS27585	0.53	3.85×10^{-3}		acyl-CoA/acyl-ACP dehydrogenase
OH82_RS27590	1.37	9.12×10^{-3}		CoA ester lyase
OH82_RS27595	0.62	4.00×10^{-4}		MaoC family dehydratase N-terminal domain-containing protein
OH82_RS28805	0.76	1.49×10^{-4}		thiamine pyrophosphate-dependent dehydrogenase E1 component subunit α
OH82_RS28810	0.94	2.97×10^{-5}		α -ketoacid dehydrogenase subunit β
OH82_RS28815	1.47	1.28×10^{-10}		acetoin dehydrogenase dihydrolipoyllysine-residue acetyltransferase subunit
OH82_RS28820	0.90	7.70×10^{-3}		glucose 1-dehydrogenase
OH82_RS07645	1.00	3.42×10^{-2}		Gfo/Idh/MocA family oxidoreductase
OH82_RS07650	0.88	1.21×10^{-5}	*eda*	bifunctional 4-hydroxy-2-oxoglutarate aldolase/2-dehydro-3-deoxy-phosphogluconate aldolase

[a] Cells were treated with 0.1 ppm ethylene or ethylene-free air for 4 h as described in the materials and methods. RNA extracted and RNAseq analysis carried out. The top 36 genes were identified as changing expression levels $|Log_2fold \geq 1|$ with an adjusted $p < 0.05$. Altered genes with an adjusted $p < 0.05$ that grouped with one or more of these 36 transcripts and occur on the same DNA strand were identified by manually examining the entire list of differentially expressed genes (Supplemental Data).
