# Supplementary material for: Ethylene signals through an ethylene receptor to modulate biofilm formation and root colonization in a beneficial plant-associated bacterium
Source: PLoS Genet. 2025 Feb 7;21(2):e1011587. doi: 10.1371/journal.pgen.1011587 (PMC11819568; doi:10.1371/journal.pgen.1011587)
Supplement: S2 Table — (PDF) [file pgen.1011587.s014.pdf]

S2 Table. Genes predicted to be co-transcribed in response to ethylene but, not included in the top 36 genes altered by ethylene.[a]

locus tag	Log_2fold change	adjusted *p*- value	gene name	annotation
OH82_RS01520	0.33	3.84×10^{-2}	*xylA*	ABC transporter permease
OH82_RS01525	0.35	5.16×10^{-3}	*xylB*	substrate-binding domain-containing protein
OH82_RS01530	0.58	1.56×10^{-4}		sugar phosphate isomerase/epimerase
OH82_RS01535	0.97	1.40×10^{-9}		Gfo/Idh/MocA family oxidoreductase
OH82_RS01540	0.69	1.80×10^{-5}		xylose isomerase
OH82_RS01545	0.53	1.16×10^{-3}		xylulokinase
OH82_RS01655	0.40	3.12×10^{-2}	*leuC*	3-isopropylmalate dehydratase large subunit
OH82_RS01660	0.44	3.74×10^{-3}	*leuD*	3-isopropylmalate dehydratase small subunit
OH82_RS01665	0.46	1.14×10^{-3}		ABC transporter substrate-binding protein
OH82_RS01670	0.53	5.18×10^{-3}		ABC transporter ATP-binding protein
OH82_RS01675	0.66	1.42×10^{-5}		ABC transporter permease subunit
OH82_RS17040	0.34	1.23×10^{-2}		isovaleryl-CoA dehydrogenase
OH82_RS17045	0.40	3.43×10^{-3}		acetyl-CoA C-acyltransferase
OH82_RS17050	0.48	3.99×10^{-4}		acyl-CoA dehydrogenase family protein
OH82_RS17055	0.29	3.10×10^{-2}		methylcrotonoyl-CoA carboxylase
OH82_RS17060	0.53	2.70×10^{-4}		enoyl-CoA hydratase/isomerase family protein
OH82_RS17065	0.79	3.08×10^{-7}		acetyl/propionyl/methylcrotonyl-CoA carboxylase subunit α
OH82_RS17070	0.39	8.79×10^{-3}		hydroxymethylglutaryl-CoA lyase
OH82_RS17075	0.30	3.59×10^{-2}		universal stress protein
OH82_RS17080	0.31	3.86×10^{-2}		electron transfer flavoprotein-ubiquinone oxidoreductase
OH82_RS28880	0.62	1.37×10^{-2}		MaoC family dehydratase
OH82_RS28885	0.65	2.52×10^{-3}		MaoC family dehydratase
OH82_RS28890	0.62	1.53×10^{-2}		enoyl-CoA hydratase/isomerase family protein
OH82_RS28895	0.45	2.71×10^{-3}		ABC transporter substrate-binding protein
OH82_RS28900	0.61	1.83×10^{-3}		branched-chain amino acid ABC transporter permease
OH82_RS28905	0.89	9.48×10^{-67}		branched-chain amino acid ABC transporter ATP-binding protein/permease
OH82_RS28910	0.60	9.96×10^{-3}		ABC transporter ATP-binding protein
OH82_RS28915	0.31	3.69×10^{-2}		SDR family oxidoreductase
OH82_RS28920	0.62	3.32×10^{-3}		AMP-binding protein

[a] Cells were treated with 0.1 ppm ethylene or ethylene-free air for 4 h as described in the materials and methods. RNA was extracted and RNAseq analysis carried out. Groups of 5 or more genes that were altered by ethylene with an adjusted *p*-value < 0.05 and that grouped on the same DNA strand were identified by manually examining the entire list of differentially expressed genes (Supplemental Data). Any groups that included genes from the top 36 genes were eliminated and included in supplemental table 1.
