# Supplementary material for: Ethylene signals through an ethylene receptor to modulate biofilm formation and root colonization in a beneficial plant-associated bacterium
Source: PLoS Genet. 2025 Feb 7;21(2):e1011587. doi: 10.1371/journal.pgen.1011587 (PMC11819568; doi:10.1371/journal.pgen.1011587)
Supplement: S3 Table — (PDF) [file pgen.1011587.s015.pdf]

S3 Table. Metabolites significantly altered by treatment with ethylene.[a]

Compound	% change from air	*p*-value
1-Methyladenosine	34%	7.65E-02
2,3-Bisphosphoglycerate	74%	1.49E-03
2,3-Dihydroxybenzoate	-55%	5.19E-02
2-hydroxyglutaric acid	145%	4.19E-03
2-Isopropylmalate	68%	5.79E-03
3-Phosphoglycerate	93%	1.63E-04
3-Phosphoserine	797%	4.25E-02
Acetylphosphate	85%	5.20E-04
ADP-glucose	40%	3.15E-02
AICAR	91%	2.77E-02
Allantoate	245%	9.26E-04
alpha-Ketoglutarate (2-oxoglutarate)	35%	4.47E-02
aminocaproic acid	63%	3.28E-04
Aminoimidazole ribotide (AIR)	65%	2.05E-02
AMP/dGMP	21%	8.87E-02
Anthranilate	-83%	4.30E-03
Arginine	45%	4.20E-04
Bis-(3',5')-cyclic diGMP	-15%	7.32E-02
Butyryl-CoA	-23%	9.50E-02
CDP	44%	4.25E-03
Citrulline	224%	1.06E-07
CMP	76%	1.00E-02
Cytidine	103%	4.25E-02
dCMP	80%	2.30E-02
Deoxyribose phosphate	26%	6.85E-02
Dephospho-CoA	195%	1.34E-03
D-Glyceraldehyde 3-phosphate	91%	1.99E-03
dTDP	29%	3.29E-02
dTMP	91%	5.97E-04
FMN	-27%	5.11E-02
Fructose 1,6-bisphosphate	357%	1.15E-03
GDP	14%	4.41E-02
Glucosamine phosphate	106%	7.00E-04
Glucose phosphate	119%	4.71E-04
Glutamate	-25%	3.94E-02
Glutathione	22%	1.66E-02
Glycinamide ribonucleotide (GAR)	1695%	1.38E-03
GMP	41%	2.76E-02
Hypoxanthine	160%	4.88E-05
IMP	87%	4.52E-04
Inosine	363%	5.08E-05
Lactate	41%	1.80E-03
Leucine/Isoleucine	63%	3.30E-04
Lysine	97%	1.98E-02
N-Acetylglutamate	34%	3.09E-03
N-Acetylglutamine	59%	1.77E-03
NADH	70%	5.97E-02
Octulose 8/1 Phosphate	95%	1.91E-03
Octulose bisphosphate	193%	8.81E-05
Ophthalmate	61%	1.66E-03
Ornithine	174%	1.91E-07
Phosphoenolpyruvate	59%	6.73E-04
Propionyl-CoA	93%	3.81E-02
Pyruvate	34%	2.10E-02
Ribose phosphate	100%	5.42E-04
Sedoheptulose 1/7-phosphate	57%	2.87E-03

Serine	-21%	3.64E-02
sn-Glycerol 3-phosphate	921%	9.14E-02
S-Ribosyl-L-homocysteine	23242%	3.95E-03
Sulfolactate	-40%	1.23E-02
Trehalose 6-phosphate	63%	5.39E-03
Tryptophan	-69%	2.91E-04
UDP-glucose	106%	5.27E-03
UMP	48%	5.14E-03
Uracil	82%	1.28E-03
Xanthine	161%	2.14E-04
Xanthosine	299%	5.98E-04
Xanthosine 5'-phosphate	85%	6.54E-03
xylose	34%	9.39E-02

[a] Cells were treated with 0.1 ppm ethylene or ethylene-free air for 24 h and prepared for untargeted metabolomics analysis as described in the materials and methods. Metabolites altered by ethylene with a $p < 0.1$ included.
