# Supplementary material for: Ethylene signals through an ethylene receptor to modulate biofilm formation and root colonization in a beneficial plant-associated bacterium
Source: PLoS Genet. 2025 Feb 7;21(2):e1011587. doi: 10.1371/journal.pgen.1011587 (PMC11819568; doi:10.1371/journal.pgen.1011587)
Supplement: S4 Table — (PDF) [file pgen.1011587.s016.pdf]

## S4 Table. Cloning primers

| Gene | Primer Sequence* |
|---|---|
| Gateway AzoEtr1 Forward | GGGGACAAGTTTGTACAAAAAAGCAGGCTTCCGCTTCGAGGGAGCGGGTGTGGACG |
| Gateway AzoEtr1 Reverse | GGGGACCACTTTGTACAAGAAAGCTGGGTGcGCTCATGCCTGACCCCGTCac |
| Gateway KanR Promoter Forward | GGGGACAAGTTTGTACAAAAAAGCAGGCTTTCATGTAGCTTGCAGTGGGCTTAC |
| Gateway KanR Promoter Reverse | GGGGACCACTTTGTACAAGAAAGCTGGGTCGCGAAACGATCCTCATCCT |
| C75A Forward | CATCCTGGCCgcCGGCACGACGC |
| C75A Reverse | AAGGCGGCGAACAGCCAG |
| D35A Forward | CATCGTGTCCgctGTGTTAACCG |
| D35A Reverse | TGCAGGGTCAGGATTTCC |
| Azorr$^{Etr1}$ with pstI Forward | ATATATCTGCAGCCATCCCTCCATTCAGGACG |
| Azorr$^{Etr1}$ with SacI Reverse | ATATATCTCGAGATCCCCTGCACCGTCCATTC |

*Lower case denotes where mutation is inserted.
