# Supplementary material for: Ethylene signals through an ethylene receptor to modulate biofilm formation and root colonization in a beneficial plant-associated bacterium
Source: PLoS Genet. 2025 Feb 7;21(2):e1011587. doi: 10.1371/journal.pgen.1011587 (PMC11819568; doi:10.1371/journal.pgen.1011587)
Supplement: S5 Table — (PDF) [file pgen.1011587.s017.pdf]

S5 Table. qPCR primers

Gene	Forward Primer	Reverse Primer
Azoetr1	GCCGTGGSSGGCATCGTCAAG	GCGCTGAAGGGCTCCGTTGGC
Azorr^{Etr1}	TTCTTTCCCGCAAGGGATAC	GCAGATCGGTGATGACGAG
OH82_RS30850	TGGCCGTAGAGGATCTCGTT	AAGGTCGTCGCTCCCTACAG
OH82_RS14895	TCATGGGATGCCTGCTGTTC	TTTTCGCGAGATAGGGGACG
